# Global net climate effects of anthropogenic reactive nitrogen

**DOI:** 10.1038/s41586-024-07714-4

**Published:** 2024-07-24

**Authors:** Cheng Gong, Hanqin Tian, Hong Liao, Naiqing Pan, Shufen Pan, Akihiko Ito, Atul K. Jain, Sian Kou-Giesbrecht, Fortunat Joos, Qing Sun, Hao Shi, Nicolas Vuichard, Qing Zhu, Changhui Peng, Federico Maggi, Fiona H. M. Tang, Sönke Zaehle

**Affiliations:** 1 Max Planck Institute for Biogeochemistry, Jena, Germany; 2 Center for Earth System Science and Global Sustainability, Schiller Institute for Integrated Science and Society, Boston College, Chestnut Hill, MA USA; 3 Department of Earth and Environmental Sciences, Boston College, Chestnut Hill, MA USA; 4 School of Environmental Science and Engineering, Nanjing University of Information Science and Technology, Nanjing, China; 5 International Center for Climate and Global Change Research, College of Forestry, Wildlife and Environment, Auburn University, Auburn, AL USA; 6 Department of Engineering and Environmental Studies Program, Boston College, Chestnut Hill, MA USA; 7 Graduate School of Agricultural and Life Sciences, University of Tokyo, Tokyo, Japan; 8 Earth System Division, National Institute for Environmental Studies, Tsukuba, Japan; 9 Department of Atmospheric Science, University of Illinois, Urbana-Champaign, Urbana, IL USA; 10 Department of Earth and Environmental Sciences, Dalhousie University, Halifax, Nova Scotia Canada; 11 Climate and Environmental Physics, Physics Institute, University of Bern, Bern, Switzerland; 12 Oeschger Centre for Climate Change Research, University of Bern, Bern, Switzerland; 13 State Key Laboratory of Urban and Regional Ecology, Research Center for Eco-Environmental Sciences, Chinese Academy of Sciences, Beijing, China; 14 Laboratoire des Sciences du Climat et de l’Environnement, LSCE-IPSL (CEA-CNRS-UVSQ), Université Paris-Saclay, Gif-sur-Yvette, France; 15 Climate and Ecosystem Sciences Division, Lawrence Berkeley National Lab, Berkeley, CA USA; 16 Department of Biology Sciences, Institute of Environment Science, University of Quebec at Montreal, Montreal, Quebec Canada; 17 School of Geographic Sciences, Hunan Normal University, Changsha, China; 18 Environmental Engineering, School of Civil Engineering, The University of Sydney, Sydney, New South Wales Australia; 19 Department of Civil Engineering, Monash University, Clayton, Victoria Australia

**Keywords:** Element cycles, Atmospheric chemistry, Ecological modelling, Climate and Earth system modelling

## Abstract

Anthropogenic activities have substantially enhanced the loadings of reactive nitrogen (Nr) in the Earth system since pre-industrial times^1,2^, contributing to widespread eutrophication and air pollution^3–6^. Increased Nr can also influence global climate through a variety of effects on atmospheric and land processes but the cumulative net climate effect is yet to be unravelled. Here we show that anthropogenic Nr causes a net negative direct radiative forcing of −0.34 [−0.20, −0.50] W m^−2^ in the year 2019 relative to the year 1850. This net cooling effect is the result of increased aerosol loading, reduced methane lifetime and increased terrestrial carbon sequestration associated with increases in anthropogenic Nr, which are not offset by the warming effects of enhanced atmospheric nitrous oxide and ozone. Future predictions using three representative scenarios show that this cooling effect may be weakened primarily as a result of reduced aerosol loading and increased lifetime of methane, whereas in particular N_2_O-induced warming will probably continue to increase under all scenarios. Our results indicate that future reductions in anthropogenic Nr to achieve environmental protection goals need to be accompanied by enhanced efforts to reduce anthropogenic greenhouse gas emissions to achieve climate change mitigation in line with the Paris Agreement.

## Abstract (web summary)

A comprehensive model framework is used to estimate the global net direct radiative forcing of anthropogenic reactive nitrogen as being about −0.34 W m^−2^, which has a cooling effect on the climate.

## Main

**Fig. 1:**
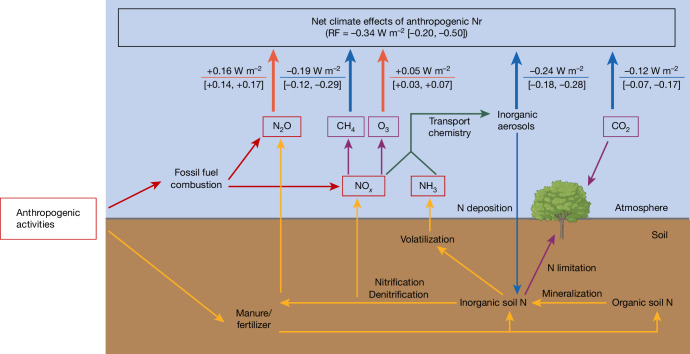
Pathways of anthropogenic Nr effects on global climate. Solid lines and arrows represent processes included in this study using a combination of terrestrial biosphere and atmospheric chemistry modelling. The direct radiative forcing estimates represent the climatic effects of anthropogenic Nr. The uncertainty range of each pathway is estimated on the basis of the 1 standard deviation across the NMIP2 ensemble members as well as the uncertainties in atmospheric chemistry (Supplementary Information Section 1.2). Orange and dark blue solid arrows on the top indicate the warming or cooling effects, respectively. The image of the tree was created using BioRender.com.

Reactive nitrogen (Nr) in the Earth system, defined as organic and inorganic forms of nitrogen (N) compounds, except the dinitrogen gas (N_2_), has increased rapidly since the industrial revolution^1^. This increase can be mainly attributed to emissions associated with anthropogenic fossil fuel combustion and fertilizer application^1,2^. Elevated concentrations of Nr induce detrimental environmental effects^3,4^, including air pollution^5^, eutrophication of surface and near-coast water^6^ and biodiversity loss^7^, but can also substantially influence climate. Specifically, the long-lived greenhouse gas nitrous oxide (N_2_O) contributes to warming of the atmosphere^8^, whereas short-lived ammonium (NH_4_^+^) and nitrate (NO_3_^−^) aerosols generated from ammonia (NH_3_) and nitrogen oxide (NO_*x*_) gases can scatter solar radiation and thereby cool the atmosphere^9–11^. NO_*x*_ furthermore plays a pivotal role in various atmospheric chemical reactions, regulating the lifetimes and thus mole fractions of other gases, such as the greenhouse gases methane (CH_4_)^12^ and ozone (O_3_)^13^. Furthermore, fertilizer application and deposition of atmospheric Nr on land and ocean can alleviate N limitation in terrestrial or marine ecosystems and facilitate carbon sequestration, thereby reducing atmospheric CO_2_ concentrations^14,15^ and exerting a cooling effect on the atmosphere (Fig. 1).

So far, the net global Nr climate effect remains unclear because of the substantial variation of individual Nr-related processes across geographic regions^16^ and the timescale dependence of the climate responses to anthropogenic Nr (ref. ^17^). An earlier study estimated a global net radiative forcing of anthropogenic Nr of −0.24 [+0.2, −0.5] W m^−2^ based only on literature review^18^. Some studies, focusing on hotspots of anthropogenic Nr, such as the United States^19^, Europe^20^ and China^21^, have also assessed the components of the regional climate effects of anthropogenic Nr based on a literature review of the sensitivities of individual processes to anthropogenic Nr inputs. However, these assessments were constrained by their focus on present-day anthropogenic Nr levels, thus neglecting the cumulative effects of long-lived greenhouse gases since the pre-industrial era. They were also limited by the extent to which they consider spatial heterogeneity and nonlinearities between the coupled biogeochemical cycles and atmospheric lifetime of the different forcers, resulting in significant uncertainties that impede attempts to extrapolate regional estimates to globe scale.

Filling these knowledge gaps requires the integration of terrestrial biogeochemistry and atmospheric chemistry to account for the intricate transformations of Nr compounds and the resulting trade-offs in the climate impacts^22^. Previous studies have demonstrated the effectiveness of both process-based terrestrial biosphere models and global chemical transport models separately in assessing the climate effects of specific anthropogenic Nr compounds or processes^9,16,23–26^. However, most of the studies associated with terrestrial Nr fluxes only relied on a single model, whereas incorporating model uncertainty is essential for a robust assessment^27^.

Here we present a comprehensive model framework to estimate the global net direct radiative forcing of anthropogenic Nr as well as the likely changes in radiative forcings in response to future changes of anthropogenic Nr inputs. First, we integrated anthropogenic emission inventories from the community emissions data system (CEDS) and eight terrestrial biosphere model outputs from the global nitrogen/N_2_O model inter-comparison project phase 2 (NMIP2)^28^ to quantify the historical anthropogenic Nr effects on terrestrial carbon sequestration, soil NH_3_ volatilization and soil NO_*x*_ and N_2_O emissions. Second, we performed a series of model experiments using box models of greenhouse gases and a global chemical transport model (GEOS-Chem) coupled to a radiative transfer module (RRTMG) to estimate the global net direct radiative forcing of anthropogenic Nr associated with each of these emission sources. Finally, we estimated how the net direct radiative forcing may respond to future scenarios of anthropogenic Nr inputs.

## Effects of anthropogenic Nr on emissions

**Fig. 2:**
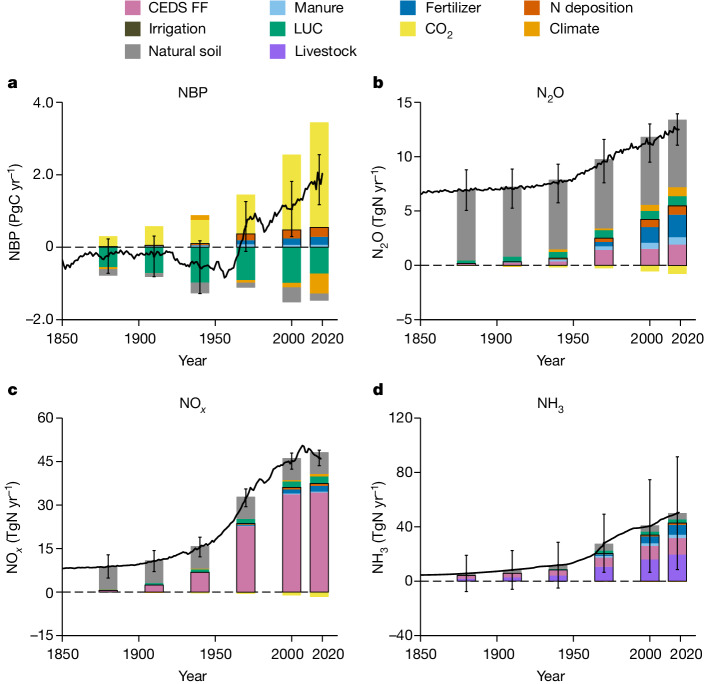
Historical Nr emissions and terrestrial carbon fluxes based on CEDS inventory and NMIP2 ensemble mean. **a**–**d**, The terrestrial NBP (**a**), N_2_O (**b**), NO_*x*_ (**c**) and NH_3_ (**d**) emissions, respectively. All of the fossil fuel sources in CEDS are indicated by the pale violet bars, whereas other colours indicate factor contributions based on the NMIP2 ensemble mean. The soil NH_3_ emissions have been scaled by the CEDS agricultural emissions (Methods). The fire emission of each component is not included in the current NMIP2 configuration. The contributions of each factor were averaged over 1880s, 1910s, 1940s, 1970s, 2000s and 2020s with a 5-year time window, in which the direct anthropogenic Nr effects are shown with a black outline. Black lines indicate the ensemble mean annual flux of each compound and the error bars indicate 1 standard deviation among different NMIP2 members. FF, fossil fuel.

We integrated results from the NMIP2 ensemble with the CEDS inventory (Methods) to comprehensively represent anthropogenic Nr effects on terrestrial carbon fluxes, N_2_O, NH_3_ and NO_*x*_ emissions (Fig. 2). Here, anthropogenic Nr sources were defined as the set of anthropogenic activities that directly add Nr into terrestrial ecosystems or the atmosphere, including manure and fertilizer application, N deposition, fossil fuel combustion and livestock NH_3_ emissions. Other anthropogenic factors in the configuration of NMIP2, for example, irrigation, land-use change (LUC), elevated CO_2_ and changing climate, also affect the global N cycle indirectly and thereby modify the response of the terrestrial biosphere to Nr additions. However, a robust identification of anthropogenic contributions from these indirect factors is not possible and therefore the influences of these indirect factors were not attributed to anthropogenic Nr effects in this study.

**Extended Data Fig. 1:**
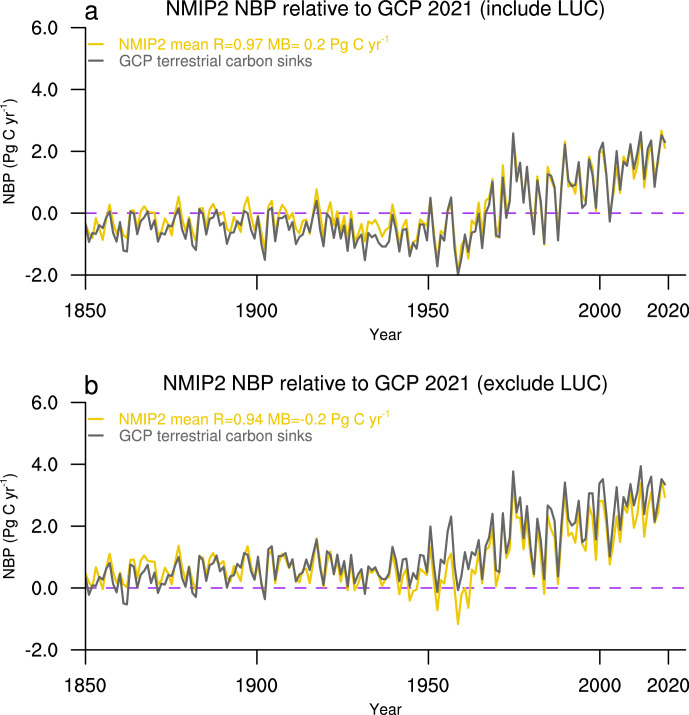
Comparison of net biome productivity (NBP) estimates from the NMIP2 ensemble and the Global Carbon Project (GCP2021). The yellow and gray lines represent the NBP of NMIP2 ensemble and GCP2021^29^ estimate, respectively. **a** and **b** show time series with and without the effect of land use change (LUC), respectively. The correlation coefficient (R) and mean bias (MB) of annual NBP between NMIP2 ensemble and GCP2021 are also given.

The net biome productivity (NBP) of the NMIP2 ensemble, which corresponds the terrestrial carbon balance, showed similar magnitudes and trends relative to Global Carbon Project 2021^29^, with the correlation coefficient of 0.94 (0.98) and mean bias of −0.1 PgC yr^−1^ (0.2 PgC yr^−1^) when excluding (including) the effects of LUC, respectively (Extended Data Fig. 1). Anthropogenic Nr, including fertilizer and manure applications and N deposition, increased terrestrial carbon sinks by 0.55 ± 0.38 PgC yr^−1^ over 2016–2020 (Fig. 2a). The N_2_O emissions from both soils and fossil fuel combustion over 2016–2020 were 12.6 ± 1.5 TgN yr^−1^, for which anthropogenic Nr contributed about 5.5 ± 0.97 TgN yr^−1^. The NMIP2 ensemble estimates of global soil N_2_O emissions induced by manure and fertilizer application, N deposition and from natural soil during 2016–2020 were 2.7 ± 0.95, 0.80 ± 0.22 and 6.2 ± 1.6 TgN yr^−1^, respectively (Fig. 2b). These estimates fall well within the uncertainty ranges of 2.5–5.8, 0.4–1.4 and 4.9–6.5 TgN yr^−1^ over 2007–2016 according to the latest N_2_O budget estimates^30^.

Anthropogenic NO_*x*_ emissions during 2016–2020 reached 46.5 ± 2.7 TgN yr^−1^, most of which were due to fossil fuel combustion, as derived from CEDS. The NMIP2 ensemble estimated that anthropogenic activities contributed about 3.1 ± 0.77 TgN yr^−1^ of the 12.2 ± 2.7 TgN yr^−1^ global soil NO_*x*_ emissions over 2016–2020 (Fig. 2c), the last of which was slightly higher than the recent estimates of about 9.5 ± 0.4 TgN yr^−1^ for 1980–2017^31^. Independent estimates of the present-day global NH_3_ emissions are highly uncertain with a range of 40–163 TgN yr^−1^ (refs. ^32–34^). This uncertainty is also reflected in the spread of the NMIP2 ensemble (Fig. 2d and Supplementary Fig. 1), which, however, showed a consistent relative imprint of agricultural fertilizer and manure applications on the trend of global NH_3_ emissions from 1850 to 2019. To derive a globally consistent time evolution of the anthropogenic Nr effect on NH_3_ emissions for our climate assessment, we adopted a conservative estimate of total NH_3_ emissions of 50.5 TgN yr^−1^ in 2019 based on CEDS inventory and applied relative contribution of anthropogenic Nr to NH_3_ emissions simulated by the NMIP2 ensemble (Methods).

## Radiative forcing from anthropogenic Nr

We next examined the net climate effects of anthropogenic Nr by combining the box-model simulated atmospheric CO_2_, N_2_O and CH_4_ concentrations and the emissions of NH_3_ and NO_*x*_, in the GEOS-Chem-RRTMG model with and without accounting for the anthropogenic Nr effect (Methods). Changes in the CH_4_ lifetime due to the effect of changing atmospheric NO_*x*_ burden on hydroxyl radical (OH) were also calculated offline using a box model (Methods). The direct radiative forcing of anthropogenic Nr for each compound was then calculated as the difference in all-sky radiative forcing at the top of the atmosphere between present-day (here defined as 2019) and pre-industrial (here defined as 1850) times. The uncertainties were estimated on the basis of the spread across the NMIP2 ensemble members as well as in atmospheric chemistry (Supplementary Information Section 1.2).

**Fig. 3:**
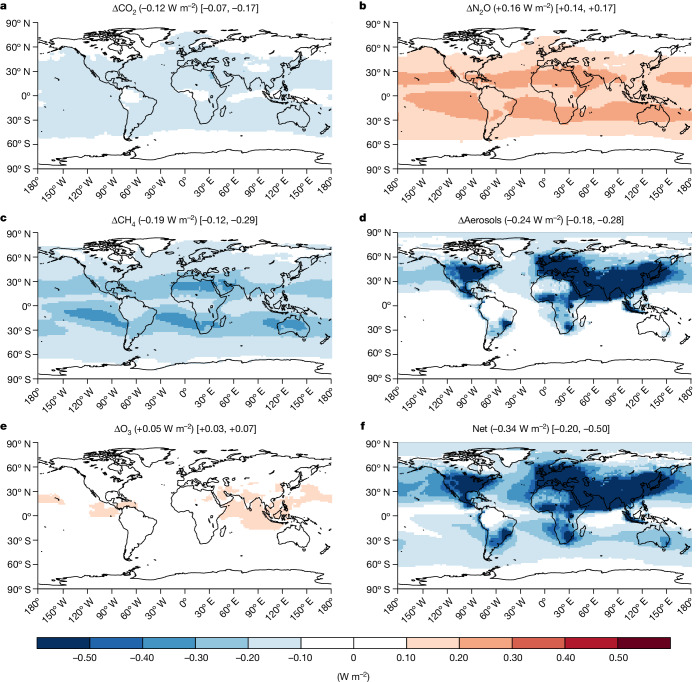
Global direct radiative forcing in 2019 induced by anthropogenic Nr. **a**–**f**, The contributions of CO_2_ (**a**), N_2_O (**b**), CH_4_ (**c**), aerosols (**d**), O_3_ (**e**) and the net effect (**f**) (that is, sum of **a**–**e**) were derived in the GEOS-Chem-RRTMG model by calculating differences in all-sky top-of-atmosphere radiative forcing between CTRL_2019 and No_allNr experiments. The radiative forcing of aerosols is the sum of the direct radiative forcing contributed by ammonium, nitrate and sulfate aerosols. Numbers in parentheses represent the global area-weighted averages, whereas numbers in the brackets indicate the uncertainty ranges based on sensitivity experiments with GEOS-Chem-RRTMG using ±1 standard deviation among NMIP2 ensembles as well as ±30% uncertainty in OH and O_3_ concentrations (Supplementary Information Section  1.2). Note the Nr effects on global CO_2_, N_2_O and CH_4_ are assumed to be evenly distributed, so that the patterns of these three greenhouse gases are mostly determined by other forcing agents, including the distribution of clouds.

**Extended Data Fig. 2:**
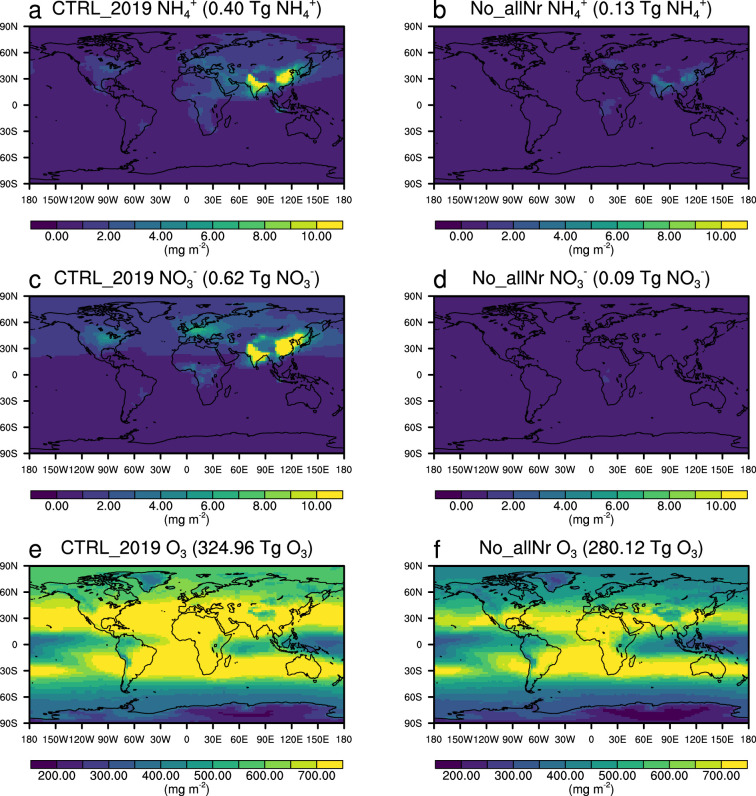
Simulated global burden of short-lived atmospheric compounds. The column burden of **a-b** ammonium aerosol, **c-d** nitrate aerosol and **e-f** O_3_ in CTRL_2019 and No_allNr experiments are given, respectively. The column burden of each compound was accumulated over the whole atmospheric column in GEOS-Chem based on the annual mean concentrations.

The net global direct radiative forcing associated with the cumulative effect of historical emissions in 2019 was estimated as −0.34 [−0.20, −0.50] W m^−2^ (Fig. 3), for which anthropogenic Nr effects on CO_2_, N_2_O, CH_4_, aerosols (including ammonium, nitrate and sulfate; Methods) and tropospheric O_3_ contributed −0.12 [−0.07, −0.17], +0.16 [+0.14, +0.17], −0.19 [−0.12, −0.29], −0.24 [−0.18, −0.28] and +0.05 [+0.03, +0.07] W m^−2^, respectively. Each component generally falls within the expected uncertainty ranges relative to previous studies focusing on individual Nr components or processes (Supplementary Information Section 3 and Supplementary Table 3). The anthropogenic Nr-induced N_2_O warming slightly outweighed the cooling effects by N-induced increases in terrestrial carbon sequestration, consistent with a previous study using one terrestrial biosphere model^9^. The enhanced NO_*x*_ emissions led to a significant cooling effect through decreasing CH_4_ lifetime and increasing aerosol burdens, whereas the negative direct radiative forcing of aerosols was unevenly distributed and prevalent in air-polluted regions such as Northern America, Western Europe and Eastern and Southern Asia. In response to the substantial NO_*x*_ increases since pre-industrial times, present-day tropospheric O_3_ was found to be enhanced across the entire simulated global grid, resulting in significant increases in global tropospheric O_3_ burden from 280.1 to 325.0 Tg (Extended Data Fig. 2). This O_3_ enhancement partly offsets the cooling climate effects from reduced CH_4_ lifetime and increased aerosol burden considering the greenhouse gas effect of O_3_.

## Splitting agricultural and other sources

To better understand the anthropogenic Nr climate effect, we further isolated the effects of agricultural and non-agricultural activities (Methods). Here, the soil emissions attributed to fertilizer and manure application were considered as agricultural sources whereas fossil fuel combustion and soil emissions attributed to changes in N deposition were regarded as non-agricultural sources. Attributing all the N deposition as non-agricultural sources omits the effect of N deposition on agricultural fluxes^35^ but this effect will be comparatively small given the much lower N deposition rates compared to agricultural fertilizer application (Fig. 2).

**Fig. 4:**
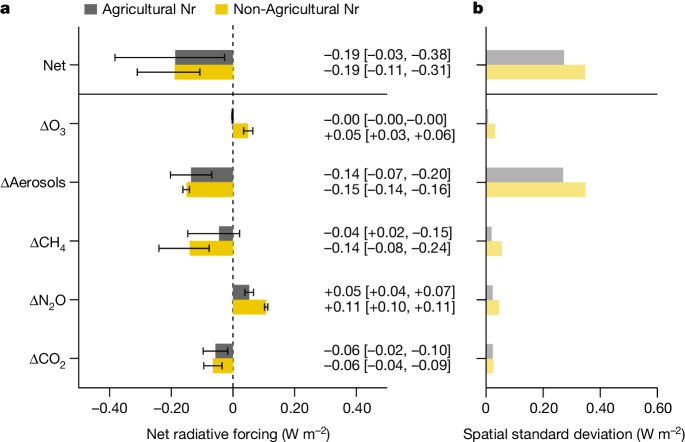
Global direct radiative forcing associated with anthropogenic Nr from agricultural and non-agricultural sources. **a**, The direct radiative forcing values are based on differences of sensitivity experiments between CTRL_2019 and No_agriNr or No_nonagriNr, respectively. The radiative forcing of aerosols is the sum of the direct radiative forcing contributed by ammonium, nitrate and sulfate aerosols. Uncertainty bars were derived from GEOS-Chem runs forced with ensemble mean ± 1 standard deviation in the NMIP2 ensemble and the associated sensitivities of radiative forcing to Nr changes (Supplementary Information Section 1.3). **b**, Spatial variation of the direct forcing effect, estimated as 1 standard deviation of direct radiative forcing across the global simulated grid.

Figure 4a showed that the net climate effects derived from agricultural and non-agricultural sources were comparable (−0.19 [−0.03, −0.38] and −0.19 [−0.11, −0.31] W m^−2^, respectively). For the agricultural sources, the net cooling effect was dominated by the direct aerosol effect, which could be attributed to the agricultural NH_3_ emissions, whereas the Nr effects of CO_2_ uptake and N_2_O emissions on the global radiative forcing compensated each other, in agreement with previous studies^9^. Conversely, NO_*x*_ emissions emitted from fossil fuel combustion dominated the net cooling effects of non-agricultural sources. Higher atmospheric NO_*x*_ burden not only induced a higher nitrate aerosol burden but also significantly decreased the atmospheric mole fraction of CH_4_ through increasing atmospheric OH. The warming effect of non-agricultural N_2_O was amplified by the synchronous decline in atmospheric CH_4_ because of their interactions in the radiative transfer^36^. We quantified this unmasking effect on the N_2_O radiative forcing by decreasing CH_4_ using a sensitivity experiment with GEOS-Chem-RRTMG (Extended Data Table 1) as a decrease in the non-agricultural N_2_O radiative forcing from +0.11 to + 0.07 W m^−2^.

Because of the large difference in lifetimes between long-lived greenhouse gases (such as CO_2_ and N_2_O) and short-lived reactive gases (such as NH_3_ and NO_x_), regional differences in the emissions matter more for the short-lived gases. As a result, the strength of the regional anthropogenic Nr climate effects shows large spatial variations (Figs. 3 and 4b). In particular, the aerosol effects tend to be strong in regions with high levels of air pollution, such as India and eastern China but show negligible radiative forcing on open ocean because of their short lifetime and limited atmospheric transport.

**Extended Data Fig. 3:**
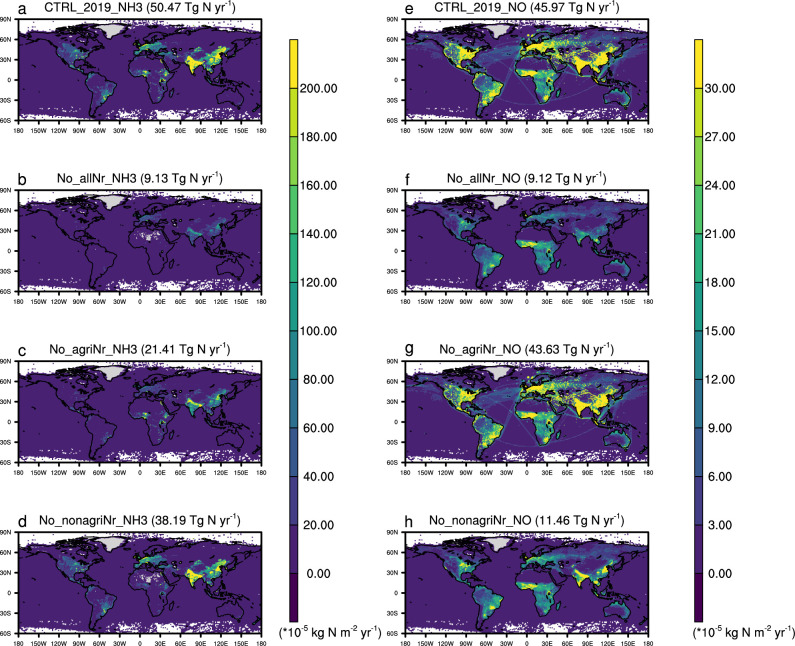
Global patterns of anthropogenic NH_3_ and NO_x_ emissions. **a-d** The NH_3_ emissions and **e-h** NO_x_ emissions in the CTRL_2019, No_allNr, No_agriNr, and No_nonagriNr sensitivity experiments, respectively, are shown. The sub-title of each panel gives the global total emissions in the year of 2019.

It should be noted that the radiative forcings attributed to agricultural and non-agricultural Nr are affected by the nonlinearity in the chemistry of aerosol formation, which results in a somewhat stronger net cooling effect from the sum of the individual effects (−0.38 W m^−2^) compared to the combined estimate (−0.34 W m^−2^ in Fig. 3f). The direct radiative forcing of nitrate aerosol is not only weakened with substantial NO_*x*_ reductions in the no_nonagriNr experiment but also reduced by the decline in the ammonium nitrate aerosol associated with significant NH_3_ emission reduction in the no_agriNr experiment (Methods; Extended Data Fig. 3 and Supplementary Table 5). The NO_*x*_ reduction further affects the concentrations of atmospheric oxidants such as O_3_ and OH and reduces the formation of sulfate aerosol in no_nonagriNr experiment (Supplementary Table 5; Methods). Nevertheless, this nonlinearity in aerosol chemistry does not influence the ranking or overall magnitude of the factors by which Nr influences radiative forcing.

## Scenarios of future Nr climate effects

**Fig. 5:**
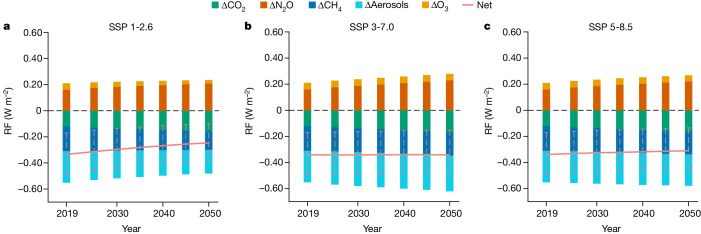
Global direct radiative forcings induced by future scenarios of anthropogenic Nr. **a**–**c**, Global direct radiative forcing relative to pre-industrial concentrations (1850) in response to changes in anthropogenic Nr inputs following SSP 1-2.6 (**a**), SSP 3-7.0 (**b**) and SSP 5-8.5 (**c**) scenarios. The net changes in radiative forcing are shown as solid orange lines. The dashed purple lines indicate no direct radiative forcing change relative to pre-industrial times. The error bars were calculated by the percentage ranges in direct radiative forcing derived from the historical estimates (Supplementary Information Section 1.3).

**Extended Data Fig. 4:**
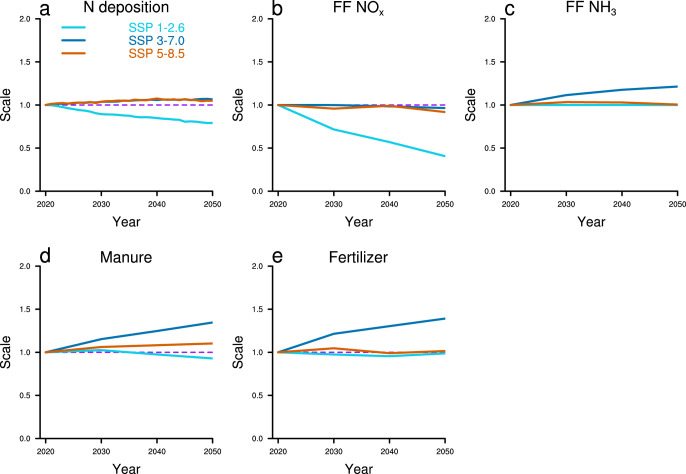
Development of global anthropogenic Nr inputs in future SSP scenarios. **a-e** indicate the changes of anthropogenic Nr inputs from N deposition, fossil fuel NO_x_, fossil fuel NH_3_, manure and fertilizer applications, respectively. For each SSP scenario, the annual scale factors were calculated by the ratios of future anthropogenic Nr to the 2019 levels, which is indicated by the dashed purple lines. The N deposition and fossil fuel data are from the input4MIPs (https://esgf-data.dkrz.de/projects/input4mips-dkrz/). Manure and fertilizer predictions are obtained from Mogollon, et al.^96^.

To illustrate the likely consequences of potential future changes in anthropogenic Nr, we next use the understanding gained in the previous section in a simplified analysis using three representative scenarios from the shared socioeconomic pathways (SSPs; Methods). The SSP 1-2.6 assumes an ‘Nr cleaner’ scenario with strong reduction in fossil-fuel-based NO_*x*_ emission but relatively unchanged magnitudes of fertilizer and manure application to meet global food demands (Extended Data Fig. 4). These Nr-related emissions changes lead to a net warming effect of +0.09 W m^−2^ by the 2050s relative to 2019 dominated by the increased CH_4_ lifetime and a decreased direct aerosol effect (Fig. 5a). In the SSP 3-7.0 scenario, the future global total fossil fuel sources of Nr remain close to the 2019 level, resulting in similar magnitude of global aerosol forcing but potentially various trends among different regions. Enhanced fertilizer and manure applications increase N_2_O emissions and lead to a stronger N_2_O warming effect of +0.06 W m^−2^ in the 2050s relative to 2019, which is compensated by the cooling effects of increased aerosol loadings (−0.03 W m^−2^ enhancement in 2050s relative to 2019) and enhanced terrestrial carbon sequestration (−0.04 W m^−2^ enhancement in the 2050s relative to 2019). However, bounding assumptions on the magnitude of N saturation (Supplementary Information Section 2.1 and Supplementary Fig. 4) suggest that carbon sequestration effect might be overestimated by about 0.02 W m^−2^. Finally, the SSP 5-8.5 scenario predicts a generally unchanged level of anthropogenic Nr compared to 2019, thus compensating changes in climate forcing. These results imply that stronger reductions in greenhouse gases emissions are required accompanied by the ‘clean-Nr’ scenario to achieve both environmental benefits and climate change mitigation.

The magnitude of the estimated radiative forcing is associated with uncertainties in each individual compound or process (Supplementary Information Section 2) but also the unavoidable ambiguity in defining the scope of anthropogenic impacts. Here we adapted a straightforward but conservative definition with only direct Nr inputs by anthropogenic activities. However, other human-induced factors, such as elevated CO_2_ and LUC, as well as the climate change and associated impacts (for example, wildfire) can have substantial impacts on biogeochemical cycling, including C^29^, water^37^ and N cycles, thus making it challenging to unambiguously identify the contributions from anthropogenic Nr. Although these indirect effects might amplify the overall climate effects of anthropogenic Nr, the NMIP2-ensemble simulations suggest that these effects on the C or N cycle are not as significant as the overall direct anthropogenic Nr effect.

In this study, several processes, including the influences of aerosols or O_3_ on terrestrial carbon fluxes, aerosol–cloud interactions, N addition effects on soil CH_4_ uptakes and N fertilization on marine biogeochemistry were not included because of the likely small effect on climate or uncertainty to quantify the global effect (Supplementary Information Section 2.5). For the effects we examined in this study, on the one hand, the future CO_2_ cooling due to CO_2_ uptake on land may be overestimated in our study because we omit the contribution of fossil-fuel-based CO_2_ emissions from N fertilizer production by the Haber–Bosch method and, more importantly, terrestrial ecosystems exposed to high chronic N additions may become N saturated within the next few decades and contribute less to terrestrial C storage (Supplementary Information Section 2.1). Uncertainties also remain in quantifying soil N_2_O, NO_*x*_ and NH_3_ emissions (Supplementary Information Sections 2.2 to 2.4). On the other hand, the negative radiative forcing of nitrate aerosol may be overestimated, as the GEOS-Chem model tends to overestimate nitrate aerosol concentrations^38–40^. Furthermore, changes in NO_*x*_ can further influence the formation of organic aerosols by altering atmospheric oxidation capacity and aerosol yields^41–43^, which are not examined in this study given the large uncertainty in simulating corresponding chemical processes. To reduce uncertainties and gain a more comprehensive understanding of potential feedbacks, the development of more integrative Earth system models including key interactions among processes of terrestrial and marine biogeochemistry, atmospheric chemistry, climate dynamics and radiative processes would be required.

Comprehensively assessing the global climate effects of anthropogenic Nr has been challenging for decades considering the complexity in atmospheric physical and chemical processes as well as the terrestrial biogeochemical cycles. Bringing together biosphere and atmospheric chemistry modelling, our results contribute to a clearer picture that at present the combined effects from short-lived and long-term Nr-related climate forcers is a global net cooling with strong regional variations. The enhanced consistency allows us to estimate the net radiative forcing of anthropogenic Nr at −0.34 [−0.20, −0.50] W m^−2^, which improves the robustness relative to the only other available estimate based on literature review alone (−0.24 [ + 0.2, −0.50] W m^−2^) (ref. ^18^). Future reductions in anthropogenic Nr will likely weaken this net cooling effect mainly through a reducing atmospheric aerosol burden and an increased CH_4_ lifetime, whereas the future effect of warming from fertilizer-induced N_2_O emissions will remain or even increase. Our findings thus imply that to alleviate the negative environmental effects of Nr without larger rates of climate change, stronger reductions in the emission of greenhouse gases CO_2_ and CH_4_ need to be implemented concurrently with Nr reductions.

## Methods

A summary for the data and methods in this study is given in Extended Data Fig. 6. Here we introduce each part in detail.

### NMIP2 multimodel dataset

The NMIP2 ensemble included eight terrestrial biosphere models with comprehensive descriptions of terrestrial carbon and nitrogen cycles, driven by harmonized climate, land use and nitrogen cycle drivers. Each NMIP2 member provided data at a spatial resolution of 0.5° × 0.5° from 11 transient, factorial simulations (Extended Data Table 2) to disentangle the contributions of N fertilizer use, manure application, N deposition, irrigation, LUC, CO_2_ elevation and climate changes from pre-industrial times (1850) to present day (2020). Climate data were generated from CRU-JRA55 6-h forcing^44^; historical CO_2_ concentrations were derived from ice core CO_2_ data and NOAA annual observations. Anthropogenic Nr deposition was generated by international global atmospheric chemistry/stratospheric processes and their role in climate chemistry–climate model initiative (https://www.sparc-climate.org/activities/ccm-initiative/). Nitrogen fertilizer and manure application data were specially generated for NMIP2 based on high-resolution (5 arcmin) harmonized data on the history of anthropogenic nitrogen inputs^45^. Land use changes were generated from land-use harmonization 2 project^46,47^, surveys by the International Fertilizer Industry Association and the Food and Agricultural Organization and the Global Livestock Impact Mapping System. For more details on NMIP2 configuration and input data, refer to refs. ^28,48^. To calculate the ensemble mean, we used output from eight models for NBP and N_2_O but could only rely on six models for soil NH_3_ and three for soil NO_*x*_ emissions, respectively (Extended Data Table 3).

### CEDS inventory

The CEDS inventory was generated by integrating existing global, regional and country-specific inventories with a consistent and reproducible methodology, representing monthly grid-level anthropogenic emissions of chemically reactive gases (for example, carbon monoxide (CO), NH_3_, NO_*x*_, sulfur dioxide and non-methane volatile organic compounds), carbonaceous aerosols (black carbon and organic carbon) and greenhouse gases (CO_2_, CH_4_ and N_2_O) from 1750 to present day (updating to the latest year)^32^. For each gas or aerosol, the anthropogenic emissions were divided into eight sectors, including non-combustion agricultural, energy transformation and extraction, industrial combustion, residential, international shipping, solvents, transportation and waste disposal. Here we accessed the CEDS data from a postprocessed version by GEOS-Chem support team, which made several modifications to fit the GEOS-Chem configurations (http://wiki.seas.harvard.edu/geos-chem/index.php/CEDS_anthropogenic_emissions).

### Integration of Nr emission data

The effect of anthropogenic fertilization, manure application, N deposition, irrigation, LUC, CO_2_ elevation and climate changes on simulations of NBP, N_2_O and NO_x_ in the NMIP2 ensemble are quantified on the basis of the differences among a series of sensitivity experiments (Extended Data Table 2). The contribution of LUC is quantified by the difference between the SH12 and SH11 experiment (rather than differences between SH1 and SH6) to avoid the confounding effects from changes in fertilizer and manure application. N_2_O and NBP fluxes are accessible for all of the eight NMIP2 members, whereas the NO_*x*_ flux is only available with CLASSIC, OCN and ORCHIDEE (Extended Data Table 3).

The NH_3_ emission estimate of 39.0 TgN yr^−1^ by the NMIP2 ensemble, which accounts for agricultural NH_3_ soil emissions but not those emissions from livestock manure, is close to the CEDS agricultural NH_3_ emissions (38.2 TgN yr^−1^) for the year 2019. However, the large intermodel variability (Supplementary Fig. 1d) makes the direct use of these simulations to quantify the anthropogenic effect susceptible to biases in individual models. Therefore, we retained the original CEDS agricultural NH_3_ emission in this study and attribute soil NH_3_ emission changes by first applying a fixed ratio (48%) on the total agricultural NH_3_ emissions in 2019, whereas the rest (52%) is led by livestock according to ref. ^35^ and then scaling the anthropogenic Nr influence on soil NH_3_ emissions according to the temporal evolution of soil NH_3_ emissions in the NMIP2 ensemble.

Finally, we integrated the CEDS anthropogenic inventory and NMIP2 multimodel data to represent the anthropogenic Nr emissions. Anthropogenic emissions from fossil fuel combustion were taken as the sum of all sectors in CEDS inventory, except for the agricultural emissions. Besides fossil fuel combustion and soil Nr emissions, we examined the biomass burning emissions of Nr based on ref. ^49^. This dataset was used to provide the historical biomass burning emissions in CMIP6 from 1850 to 2015 and showed similar magnitude and variabilities as GFED4.1 inventory in the past decades. The historical annual biomass burning N_2_O emissions were used to establish N_2_O box model (see below). However, because the biomass burning emissions of NO_*x*_ and NH_3_ showed little differences between the present day and pre-industrial period, here we neglected such differences and used the same present-day biomass burning emissions in all the GEOS-Chem experiments.

### CO_2_, N_2_O and CH_4_ box models

To estimate the effects of anthropogenic Nr on atmospheric CO_2_ concentrations, we used atmospheric box models based on the framework of ref. ^9^. The changes in atmospheric CO_2_ concentrations induced by anthropogenic Nr effects on terrestrial carbon fluxes were represented by:

1 $$\Delta {{\rm{C}}{\rm{O}}}_{2}=-\mathop{\sum }\limits_{yr=1850}^{2019}({{\rm{N}}{\rm{B}}{\rm{P}}}_{{\rm{f}}{\rm{e}}{\rm{r}}{\rm{t}}{\rm{i}}{\rm{l}}{\rm{i}}{\rm{z}}{\rm{e}}{\rm{r}},{\rm{y}}{\rm{r}}}+{{\rm{N}}{\rm{B}}{\rm{P}}}_{{\rm{m}}{\rm{a}}{\rm{n}}{\rm{u}}{\rm{r}}{\rm{e}},{\rm{y}}{\rm{r}}}+{{\rm{N}}{\rm{B}}{\rm{P}}}_{{\rm{N}}{\rm{d}}{\rm{e}}{\rm{p}},{\rm{y}}{\rm{r}}})\times \frac{\alpha }{{\delta }_{{{\rm{C}}{\rm{O}}}_{2}}}$$

where $\Delta {{\rm{CO}}}_{2}$ indicates changes in atmospheric CO_2_ concentrations (ppmv) from 1850 to 2019 because of anthropogenic Nr. The accumulated NBP induced by fertilizer and manure applications and N deposition during 1850–2019 was calculated from NMIP2 ensemble mean (Extended Data Table 2). The ${\delta }_{{{\rm{CO}}}_{2}}$ was 2.12 PgC ppmv^−1^ following ref. ^50^. The partitioning constant *α* accounting for the ocean-borne fraction of atmospheric CO_2_ increase was determined to be 0.61 given the historical (1850–2019) increases in the atmosphere (235 PgC) and ocean (150 PgC) carbon estimated from the global carbon budget^29^.

**Extended Data Fig. 5:**
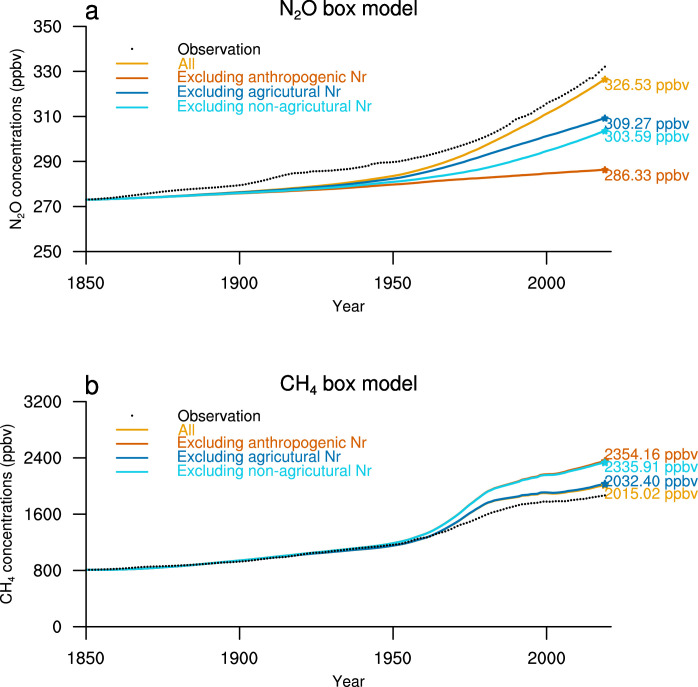
Global N_2_O and CH_4_ concentrations during 1850–2019 based on the box models. **a** N_2_O concentration; and **b** CH_4_ concentration. The black dots indicate the historical annual N_2_O and CH_4_ concentrations, as observed from ice core, firn and atmospheric measurements^101^. Lines with different colors represent the different SSP scenarios. Concentration values indicate the global mean concentration in the year of 2019 derived from the box models.

**Extended Data Fig. 6:**
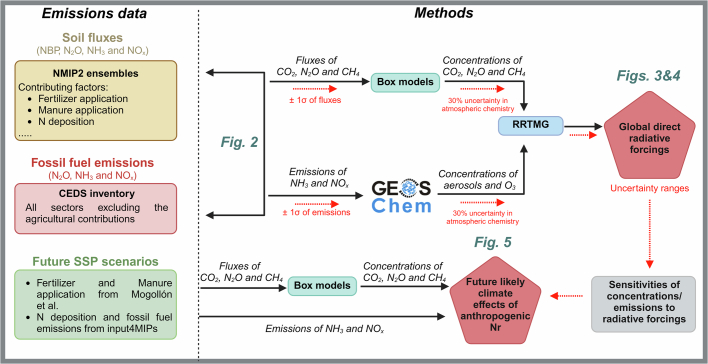
Schematic workflow summary of this study. Solid black arrows indicate main methods or data, as described in Methods. Dashed red arrows indicate the uncertainty analysis and the associated sensitivities to radiative forcing (see SI text S1). The main figures in this study are highlighted accordingly with the figure indexes. This figure is created with BioRender.com.

The N_2_O box model was also based on ref. ^9^:

2 $$\frac{{\rm{d}}{[{{\rm{N}}}_{2}{\rm{O}}]}_{{\rm{yr}}}}{{\rm{d}}t}=\frac{{{{\rm{N}}}_{2}{\rm{O}}}_{{\rm{FF}}}+{{{\rm{N}}}_{2}{\rm{O}}}_{{\rm{soil}}}+{{\rm{N}}}_{2}{{\rm{O}}}_{{\rm{BB}}}+{{{\rm{N}}}_{2}{\rm{O}}}_{{\rm{AREC}}}+{{{\rm{N}}}_{2}{\rm{O}}}_{{\rm{chem}}}+{{{\rm{N}}}_{2}{\rm{O}}}_{{\rm{NREC}}}+{{{\rm{N}}}_{2}{\rm{O}}}_{{\rm{ocean}}}}{{\delta }_{{{\rm{N}}}_{2}{\rm{O}}}}-\frac{{[{{\rm{N}}}_{2}{\rm{O}}]}_{{\rm{yr}}}}{\tau }$$

where the $\frac{{\rm{d}}{[{{\rm{N}}}_{2}{\rm{O}}]}_{{\rm{yr}}}}{{\rm{d}}t}$ was the annual increasing rate of atmospheric N_2_O concentrations at the yr year. N_2_O sources from fossil fuel (FF) combustion, soil, biomass burning (BB), anthropogenic emissions from river, estuaries and coastal zones (AREC), atmospheric chemistry, natural emissions from river, estuaries and coastal zones (NREC) as well as open ocean were summarized in Extended Data Table 4 (refs. ^9,49,51,52^). The ${\delta }_{{{\rm{N}}}_{2}{\rm{O}}}$ was set as 4.8 TgN ppbv^−1^ following ref. ^9^. The [N_2_O]_yr_ indicated the surface atmospheric N_2_O concentrations at the yr year and $\tau$ was the perturbation lifetime of atmospheric N_2_O, taken as 116 years (ref. ^52^). The simulated global surface N_2_O concentrations were shown in Extended Data Fig. 5a.

We used a CH_4_ box model described by ref. ^53^ to examine the effects of changes in NO_*x*_ emissions on CH_4_ concentrations due to changed atmospheric OH concentrations:

3 $$\frac{{\rm{d}}\,{[{{\rm{CH}}}_{4}]}_{{\rm{yr}}}}{{\rm{d}}t}=-\frac{1}{{\tau }_{{{\rm{CH}}}_{4}}}{\left[{{\rm{CH}}}_{4}\right]}_{{\rm{yr}}}+\frac{{E}_{{{\rm{CH}}}_{4},{\rm{yr}}}}{{\delta }_{{{\rm{CH}}}_{4}}}$$

where [CH_4_]_yr_ indicated the global mean CH_4_ concentrations at the yr year. ${E}_{{{\rm{CH}}}_{4},{\rm{yr}}}$ was the total CH_4_ emissions at the yr year, which was calculated by summing CH_4_ emissions by anthropogenic activities based on CEDS inventory, biomass burning emissions based on ref. ^49^ and natural sources with an estimate of 230 Tg yr^−1^. The ${\delta }_{{{\rm{CH}}}_{4}}$ was set as 2.78 Tg ppb^−1^ (ref. ^54^). The CH_4_ lifetime ${\tau }_{{{\rm{CH}}}_{4}}$ was estimated by:

4 $$\frac{1}{{\tau }_{{{\rm{CH}}}_{4}}}=\frac{1}{{\tau }_{{\rm{OH}}}}+\frac{1}{{\tau }_{{\rm{strat}}}}+\frac{1}{{\tau }_{{\rm{soil}}}}+\frac{1}{{\tau }_{{\rm{trop}}-{\rm{cl}}}}$$

where ${\tau }_{{\rm{strat}}}$, ${\tau }_{{\rm{soil}}}$ and ${\tau }_{{\rm{trop}}-{\rm{cl}}}$ were set as constant numbers of 120, 150 and 200 years, respectively, to represent CH_4_ lifetime led by stratospheric loss, soil uptake and tropospheric chlorine reactions. Parameter ${\tau }_{{\rm{OH}}}$ was the CH_4_ lifetime due to the OH oxidation, which was calculated by:

5 $$\frac{1}{{\tau }_{{\rm{OH}}}}=\frac{1}{{\tau }_{{\rm{OH}}}^{0}}\times \left({\left(\frac{{[{{\rm{CH}}}_{4}]}_{{\rm{yr}}}}{{[{{\rm{CH}}}_{4}]}_{0}}\right)}^{{S}_{{\rm{OH}}}}\times {e}^{({S}_{{{\rm{NO}}}_{x}}\times \Delta {E}_{{{\rm{NO}}}_{x}}+{S}_{{\rm{CO}}}\times \Delta {E}_{{\rm{CO}}}+{S}_{{\rm{VOC}}}\times \Delta {E}_{{\rm{VOC}}})}+{S}_{T}\times \Delta T\right)$$

where ${\tau }_{{\rm{OH}}}^{0}$ and ${\left[{{\rm{CH}}}_{4}\right]}_{0}$ were the references of CH_4_ lifetime and concentrations. Here we selected the year of 2005 as the reference year with ${\tau }_{{\rm{OH}}}^{0}$ of 11.17 years and ${\left[{{\rm{CH}}}_{4}\right]}_{0}$ of 1,783.36 ppb. The sensitivity factor *S*_OH_ was −0.31 following ref. ^55^. ${S}_{{{\rm{NO}}}_{x}}$, *S*_CO_ and *S*_VOC_ were set as 0.0042 (Tg[N] yr^−1^)^−1^, −0.000105 (Tg[CO] yr^−1^)^−1^ and −0.000315 (Tg[VOC] yr^−1^)^−1^, respectively, following Table 4.11 of ref. ^56^. The emission changes in NO_*x*_ (${\Delta E}_{{{\rm{NO}}}_{x}}$), CO (${\Delta E}_{{\rm{CO}}}$) and volatile organic compounds (VOC) (${\Delta E}_{{\rm{VOC}}}$), which included changes in anthropogenic emissions from CEDS and biomass burning emissions from ref. ^49^, were calculated by the differences between the yr year and the reference year (2005), respectively. The temperature effects on atmospheric CH_4_ loss rates were expressed by multiplying factor *S*_*T*_ of 0.0316 K^−1^ (ref. ^56^) and changes in global surface mean temperature $\triangle T$ relative to the reference year (2005). The simulated global mean surface CH_4_ concentrations were shown in Extended Data Fig. 5b.

### The GEOS-Chem-RRTMG model and sensitivity experiments

We used the state-of-art global three-dimensional chemical transport model GEOS-Chem (v.12.0.0) with a fully coupled NO_*x*_–O_*x*_–hydrocarbon–aerosol chemistry mechanism^57–60^ to simulate NH_3_ and NO_*x*_ concentration and associated aerosol loadings and O_3_ at a horizontal resolution of 2° latitude $\times$ 2.5° longitude and a vertical resolution of 47 layers from surface to 0.1 hPa level. The photolysis rates were computed by Fast-JX scheme^58^. Aerosol concentrations were calculated online by the ISORROPIA II package^61^. Version two of modern era retrospective-analysis for research and application (MERRA2) assimilated meteorological data was used to drive the GEOS-Chem model. Atmospheric concentrations of the long-lived greenhouse gases CO_2_, CH_4_ and N_2_O were derived from simple atmospheric box models (see above). On the basis of the simulated concentrations of tracers, we diagnosed direct radiative forcing of Nr-related compounds using the offline RRTMG in GEOS-Chem^62^. The annual-mean direct radiative forcing in the year 2019 was estimated from a year-long simulation after a 6 month spin-up period. In particular, GEOS-Chem fully considers the nonlinearity of inorganic aerosol chemistry, in which sulfate aerosol has higher priority than nitrate aerosol in aerosol formation when ammonia gas is limited in the atmosphere. Changes in the atmospheric NO_*x*_ loading can also affect oxidation of sulfur dioxide by perturbating atmospheric oxidants, such as O_3_ and OH. As a result, the sulfate aerosol loadings could also be perturbed by changes in NO_*x*_ emissions, despite the fact that the sulfur dioxide emissions are identical in all our experiments. We thus use the sum of direct radiative forcing of ammonium (NH_4_^+^), nitrate (NO_3_^−^) and sulfate (SO_4_^2−^) aerosols to represent the aerosol climate effects induced by anthropogenic Nr.

We designed four sensitivity experiments to isolate the anthropogenic Nr effects on climate, in which each experiment was driven by the same meteorological forcing but with different NH_3_ and NO_*x*_ emissions as well as CO_2_, N_2_O and CH_4_ concentrations. The NH_3_ and NO_*x*_ emissions in each experiment are given in Extended Data Fig. 3, whereas CO_2_, N_2_O and CH_4_ concentrations were summarized in Extended Data Table 1. An extra sensitivity experiment, which followed No_nonagriNr run but assumed CH_4_ concentrations as in the CTRL run, was designed to quantify the effect of changes in CH_4_ concentrations on the radiative forcing of N_2_O due to non-agricultural emission changes. We estimated the uncertainty in the radiative forcing estimates by propagating the variation across NMIP2 ensemble projections into atmospheric concentrations and thus radiative forcing. The full uncertainty analysis and uncertainty discussions are detailed in the Supplementary Information and rely on refs. ^9–11,18,28,32–35,52,62–95^.

### Linear extrapolation of climate effects under the SSP scenarios

We extrapolated the future climate effects due to changes in anthropogenic Nr under three representative SSP scenarios (SSP 1-2.6, 3-7.0 and 5-8.5). The future fossil fuel emissions and N deposition were from the input4MIPs dataset (https://esgf-data.dkrz.de/projects/input4mips-dkrz/). Future fertilizer and manure applications were based on the IMAGE predictions until 2050^96^. To maintain consistency in this study, the future Nr-related sources were scaled to 2019 levels for each dataset (Extended Data Fig. 4). Because the future fossil-fuel-based emission of N_2_O is not included in input4MIPs, the future development of this source of N_2_O was scaled to the future development of fossil-fuel-based NO_*x*_.

To estimate the magnitude of climate effects of anthropogenic Nr under the SSP scenarios, we built a simple linear framework based on the following assumptions. (1) The change in radiative forcing of atmospheric greenhouse gas attributable to Nr-related changes was linearly related to their change in atmospheric concentrations, whereas the direct radiative forcing of short-lived gases or aerosols was linearly related to the total emissions of precursors^11,97,98^ at the corresponding year. (2) The effects of anthropogenic Nr on soil–gas fluxes were linearly determined by anthropogenic Nr addition, including both fertilizer/manure application and N deposition. Then a simple model was established based on the GEOS-Chem diagnosed direct radiative forcing of individual compound to calculate the radiative forcing relative to 1850:

6 $$\begin{array}{l}{{\rm{RF}}\_{\rm{Nr}}\_{\rm{CO}}}_{{\rm{2\; yr}}}\,=\,{{\rm{RF}}\_{\rm{Nr}}\_{\rm{CO}}}_{2}\,2019\\ \,\,\,\,\,\,\,\,+\mathop{\sum }\limits_{{\rm{yr}}=2020}^{t}({{\rm{NBP}}}_{{\rm{fertilizer}},{\rm{yr}}}+{{\rm{NBP}}}_{{\rm{manure}},{\rm{yr}}}+{{\rm{NBP}}}_{{\rm{Ndep}},{\rm{yr}}})\\ \,\,\,\,\,\,\,\,\times \frac{\alpha }{{\delta }_{{{\rm{CO}}}_{2}}}\,\times {S}_{{{\rm{CO}}}_{2}}\end{array}$$

7 $${{\rm{RF}}\_{\rm{Nr}}\_{{\rm{N}}}_{2}{\rm{O}}}_{{\rm{yr}}}={{\rm{RF}}\_{{\rm{Nr}}\_{\rm{N}}}_{2}{\rm{O}}}_{2019}+({\left[{{\rm{N}}}_{2}{\rm{O}}\right]}_{{\rm{yr}}}-{\left[{{\rm{N}}}_{2}{\rm{O}}\right]}_{2019})\times {S}_{{{\rm{N}}}_{2}{\rm{O}}}$$

8 $${\rm{RF}}\_{\rm{Nr}}\_{{\rm{CH}}}_{{\rm{4\; yr}}}={\rm{RF}}\_{{\rm{Nr}}\_{\rm{CH}}}_{{\rm{4\; 2019}}}+({[{{\rm{CH}}}_{4}]}_{{\rm{yr}}}-{[{{\rm{CH}}}_{4}]}_{2019})\times {S}_{{{\rm{CH}}}_{4}}$$

9 $${{\rm{RF}}\_{\rm{Nr}}\_{\rm{aerosol}}}_{{\rm{yr}}}=\frac{{{\rm{NO}}}_{x{\rm{yr}}}+{{\rm{NH}}}_{3{\rm{yr}}}}{{{\rm{NO}}}_{x2019}+{{\rm{NH}}}_{{\rm{3\; 2019}}}}{\times {\rm{RF}}\_{\rm{Nr}}\_{\rm{aerosol}}}_{2019}$$

10 $${{\rm{RF}}\_{\rm{Nr}}\_{\rm{O}}}_{3{\rm{yr}}}=\frac{{{\rm{NO}}}_{x{\rm{yr}}}}{{{\rm{NO}}}_{x2019}}\times {{\rm{RF}}\_{\rm{Nr}}\_{\rm{O}}}_{3{\rm{2019}}}$$

Where the RF_Nr_CO_2 yr_, RF_Nr_N_2_O_yr_, RF_Nr_CH_4 yr_, RF_Nr_aerosol_yr_ and RF_Nr_O_3 yr_ represent the direct radiative forcing associated with anthropogenic Nr of each gas at the yr year relative to 1850. The values in 2019 were derived from the differences between CTRL_2019 and No_allNr experiments (−0.12 W m^−2^, +0.16 W m^−2^, −0.19 W m^−2^, −0.24 W m^−2^ and +0.05 W m^−2^, respectively; Fig. 3). The sensitivities (${S}_{{{\rm{CO}}}_{2}}$, ${S}_{{{\rm{N}}}_{2}{\rm{O}}}$ and ${S}_{{{\rm{CH}}}_{4}}$) of radiative forcing to greenhouse gas concentrations were derived from the other eight GEOS-Chem sensitivity experiments (Supplementary Information Section 1.3 and Supplementary Table 2).

In particular, we calculated the reduction effect as follows:

1. NBP_fertilizer,yr_, NBP_manure,yr_ and NBP_Ndep,yr_ represented the NBP contributed by fertilizer, manure and N deposition in the yr year, which is calculated by multiplying the NMIP2 ensemble mean present-day (average of 2015–2019) contributions and the corresponding scaling factors in Extended Data Fig. 4.
2. The N_2_O and CH_4_ concentrations in the yr year (${[{{\rm{N}}}_{2}{\rm{O}}]}_{{\rm{yr}}}$ and ${[{{\rm{CH}}}_{4}]}_{{\rm{yr}}}$) were derived by the simple N_2_O and CH_4_ box models (equation (2) and equations (3)–(5) starting from ${[{{\rm{N}}}_{2}{\rm{O}}]}_{2019}$ (N_2_O concentrations in CTRL_2019 experiments) and ${[{{\rm{CH}}}_{4}]}_{2019}$ (CH_4_ concentrations in CTRL_2019 experiments), respectively. N_2_O (in N_2_O box model) and NO_*x*_ (in CH_4_ box model) emissions from both fossil fuel combustion and anthropogenic Nr-induced soil emissions were reduced relative to emissions in 2019 with the scaling factors accordingly (Extended Data Fig. 4), whereas the other sources were kept the same as 2019.
3. For short-lived compounds (aerosols and O_3_), NO_*x* yr_ (or NH_3 yr_) indicated the NO_*x*_ (or NH_3_) emissions from both fossil fuel and soil by applying the scaling factors on each sector (Extended Data Fig. 4) in the yr year.

## Online content

Any methods, additional references, Nature Portfolio reporting summaries, source data, extended data, supplementary information, acknowledgements, peer review information; details of author contributions and competing interests; and statements of data and code availability are available at 10.1038/s41586-024-07714-4.

## Supplementary information

Supplementary Information Supplementary Sections 1–3, Tables 1–5, Figs. 1–5 and reference. Supplementary Section 1 introduces the methods to assess the uncertainty ranges in the climate effects of anthropogenic Nr; Section 2 discusses the main uncertainties in this study; and Section 3 compares our estimates on each Nr-related facet with previous individual studies.

Peer Review File

## Figures and Tables

**Extended Data Table 1:**
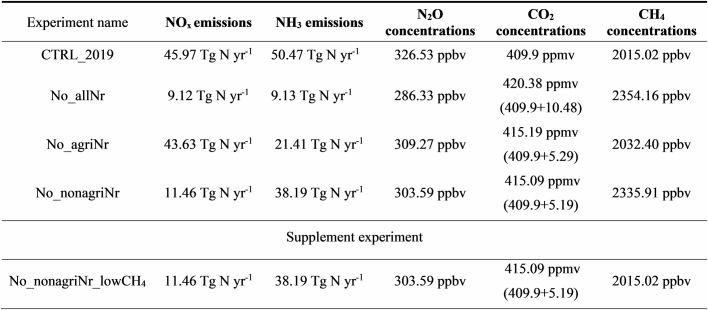
Summary of the GEOS-Chem sensitivity experiments

The global NO_x_ and NH_3_ emissions are derived by integrating CEDS and NMIP2 ensembles. Atmospheric concentrations of N_2_O, CO_2_ and CH_4_ are retrieved by the box models. The CTRL_2019 experiment includes all anthropogenic Nr sources; The No_allNr experiment excludes anthropogenic Nr sources of fossil fuel, fertilizer and manure application and N deposition; The No_agriNr experiment excludes anthropogenic Nr sources of fertilizer and manure application; The No_agriNr experiment excludes anthropogenic Nr sources of fossil fuel and N deposition. The livestock NH_3_ emission is attributed as agricultural sources.

**Extended Data Table 2:**
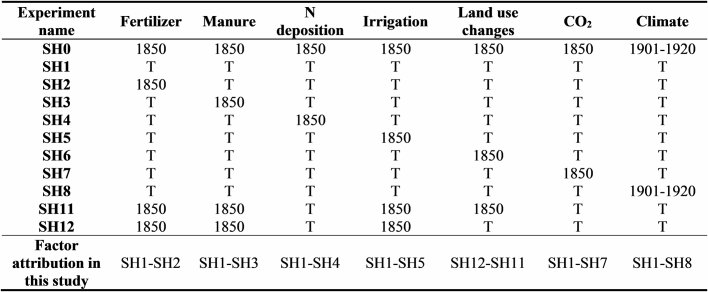
Experiment configurations in NMIP2

The letter ‘T’ indicates a transient change as the forcing data from 1850 to 2020. The 1850 or 1901–1920 indicates that the corresponding forcing was fixed in this year or time periods. Climate over 1850–1900 were repeated by 1901–1920 for all experiments due to the missing of data. The last row of the table indicates how the factorial contribution for each factor in this study is calculated from the differences between corresponding experiments.

**Extended Data Table 3:**
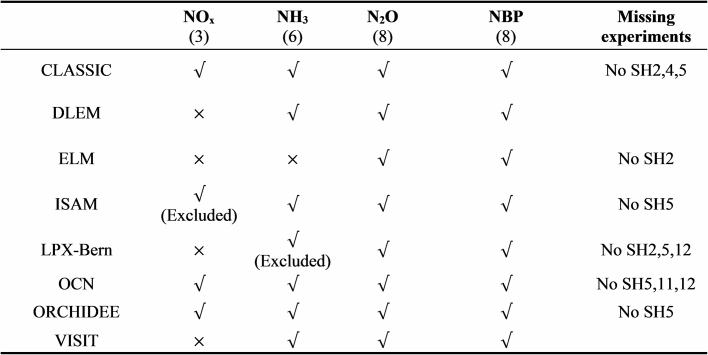
Summary of the eight terrestrial biosphere models in NMIP2 used in this study

Some of the models failed to finish all experiments, which were indicated in the last column. Soil NH_3_ volatilization of LPX-Bern was excluded from the integration of NMIP2 and CEDS since it failed to show factor contributions. Soil NO_x_ emissions simulated by ISAM was also excluded due to the unreasonably high magnitude. The number of models that we finally used to drive GEOS-Chem model for the corresponding variable was indicated in the top row.

**Extended Data Table 4:**
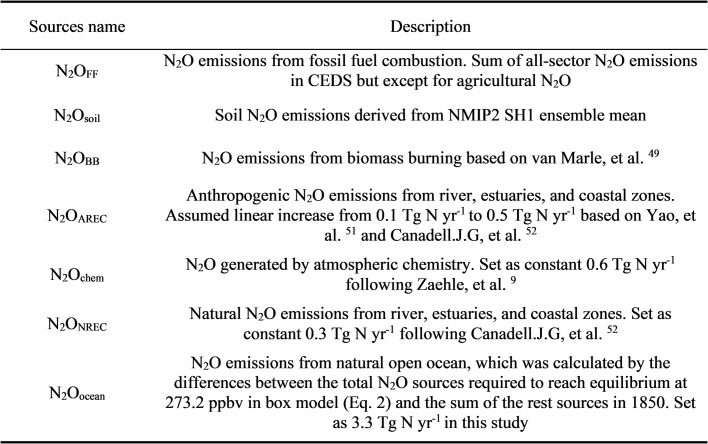
Summary of N_2_O sources applied in the box model

All N_2_O fluxes represented an annual time series from 1850 to 2019.

## Data Availability

The CEDS inventory used in GEOS-Chem can be downloaded at https://ftp.as.harvard.edu/gcgrid/data/ExtData/HEMCO/CEDS/. The NMIP2 model outputs and the GEOS-Chem outputs in this study are available at Zenodo (10.5281/zenodo.10032973)^99^. The base maps in all figures are based on the default global map in the NCAR Command Language (NCL).

## Code availability

The GEOS-Chem-RRTMG source codes can be accessed at https://github.com/geoschem/geos-chem. Data analysis and visualization are conducted by NCL. Scripts are available at Zenodo (10.5281/zenodo.11179126)^100^.
