# Supplementary material for: Global net climate effects of anthropogenic reactive nitrogen
Source: Nature. 2024 Jul 24;632(8025):557–63. doi: 10.1038/s41586-024-07714-4 (PMC11324526; doi:10.1038/s41586-024-07714-4)
Supplement: Supplementary file 1 — Supplementary Sections 1–3, Tables 1–5, Figs. 1–5 and reference. Supplementary Section 1 introduces the methods to assess the uncertainty ranges in the climate effects of anthropogenic Nr; Section 2 discusses the main uncertainties in this study; and Section 3 compares our estimates on each Nr-related facet with previous individual studies. [file 41586_2024_7714_MOESM1_ESM.docx]

*Supplementary Information for*

**Global net climate effects of anthropogenic reactive nitrogen**

Cheng Gong^1*^, Hanqin Tian^2,3^, Hong Liao^4^, Naiqing Pan^2, 5^, Shufen Pan^6,2^, Akihiko Ito^7,8^, Atul K. Jain^9^, Sian Kou-Giesbrecht^10^, Fortunat Joos^11,12^, Qing Sun^11,12^, Hao Shi^13^, Nicolas Vuichard^14^, Qing Zhu^15^, Changhui Peng^16,17^, Federico Maggi^18^, Fiona H.M. Tang^19^ and Sönke Zaehle^1^

^1^Max Planck Institute for Biogeochemistry, Jena, Germany

^2^Center for Earth System Science and Global Sustainability, Schiller Institute for Integrated Science and Society, Boston College, Chestnut Hill, MA, USA

^3^Department of Earth and Environmental Sciences, Boston College, Chestnut Hill, MA, USA^4^School of Environmental Science and Engineering, Nanjing University of Information Science and Technology, Nanjing, China

^5^International Center for Climate and Global Change Research, College of Forestry, Wildlife and Environment, Auburn University, Auburn, AL, USA

^6^Department of Engineering and Environmental Studies Program, Boston College, Chestnut Hill, MA 02467, USA

^7^Graduate School of Life and Agricultural Sciences, University of Tokyo, Tokyo, Japan

^8^Earth System Division, National Institute for Environmental Studies, Tsukuba, Japan

^9^Department of Atmospheric Science, University of Illinois, Urbana-Champaign, Urbana, 61801, IL, USA

^10^Department of Earth and Environmental Sciences, Dalhousie University, Halifax, NS, Canada

^11^Climate and Environmental Physics, Physics Institute, University of Bern, Bern, Switzerland

^12^Oeschger Centre for Climate Change Research, University of Bern, Bern, Switzerland

^13^State Key Laboratory of Urban and Regional Ecology, Research Center for Eco-Environmental Sciences, Chinese Academy of Sciences, Beijing 100085, China

^14^Laboratoire des Sciences du Climat et de l'Environnement, LSCE-IPSL (CEA-CNRS-UVSQ), Université Paris-Saclay 91191 Gif-sur-Yvette, France

^15^Climate and Ecosystem Sciences Division, Lawrence Berkeley National Lab, 1 Cyclotron Road, Berkeley, CA 94720, USA

^16^Department of Biology Sciences, Institute of Environment Science, University of Quebec at Montreal, Montreal, QC H3C 3P8, Canada

^17^School of Geographic Sciences, Hunan Normal University, Changsha 410081, China

^18^Environmental Engineering, School of Civil Engineering, The University of Sydney, Sydney, NSW, Australia

^19^Department of Civil Engineering, Monash University, Clayton, VIC, Australia

Correspondence to: Cheng Gong (cgong@bgc-jena.mpg.de)

**Supplementary Information**

This supplementary information includes 3 sections of texts, 5 Tables and 5 Figures. In the texts, Sect. S1 introduced the methods to assess the uncertainty ranges in the climate effects of anthropogenic Nr. Sect. S2 discussed the main uncertainties in this study. Sect. S3 compared our estimates on each Nr-related facet with previous individual studies.

**S1. Uncertainties analysis**

S1.1 Uncertainties in the NMIP2 model ensemble

The NMIP2 ensemble consists of eight terrestrial biosphere models with fully-coupled C and N cycles. While the models represent a comprehensive budget of the terrestrial N cycle and generally simulate global fluxes within the range of published estimates, the models are based on diverging representations of key processes, including biological N fixation, N mineralization, nitrification and denitrification, which contributes to inter-model variability as revealed by the substantial standard deviation of the mean. The models represent the individual sensitivities of simulated N_2_O to elevated CO_2_, warming and changes in wetness broadly in agreement with observations, but given the lack of suitable observations, the interactions among different environmental forcing remains insufficiently evaluated. Tian, et al. ^1^ highlight the representation of the human nitrogen management practices in agriculture and the N effects of seasonal freezing-thaw in permafrost as key weaknesses of these models. However, even with these limitations, terrestrial biosphere models are still the most straightforward and powerful tools to isolate different sectors of anthropogenic Nr and understand their substantial influences on the Nr-related gas fluxes.

S1.2 Quantification of climate effects

We analyzed the uncertainty in the estimates of climate forcing from anthropogenic Nr in term of the spread across the NMIP2 ensemble members as well as uncertainties in atmospheric chemistry.

Figure S2 shows the contributions of anthropogenic Nr on terrestrial carbon fluxes and terrestrial emissions of N_2_O, NO_x_ and NH_3_ as simulated by the NMIP2 ensemble, where the shaded area reflects ±one standard deviation across the NMIP2 models. These uncertainties were already represented in the error bars of Fig. 2 and reflect relatively large uncertainties in the Nr effects on NBP and NH_3_ emissions with about 0.5±0.35 Pg C yr^-1^ and 43.4±18.8 Tg N yr^-1^ in 2019, respectively, while the NO_x_ emission is less uncertain due to the dominant roles of fossil fuel combustion: in fact, the soil-based NO_x_ emissions derived from anthropogenic Nr inputs remained uncertain with 3.13±0.68 Tg N yr^-1^ in 2019.

Soil-based NO_x_ emissions contribute a small part in the global NO_x_ emissions and therefore also its uncertainty. The IPCC AR6 report estimated the uncertainties in fossil fuel derived NO_x_ emissions at about 10%^2^. However, relative to the consistently similar magnitudes and trends in both bottom-up and top-down estimates of present-day NO_x_ emissions^3-5^, the indirect effect of NO_x_ on atmospheric OH and therefore the atmospheric O_3_ and CH_4_ have relatively higher uncertainties due to the non-linear atmospheric chemistry process. To approximate the impacts of uncertainty in the atmospheric processing of NO_x_ on our estimates of the NO_x_-related climate effects, we assigned ±30% uncertainty for the global OH estimates in the CH_4_ box model (derived from Murray, et al. ^6^), which contributes to a much higher variation in atmospheric CH_4_ compared to direct changes in NO_x_ emissions applied in this study. We also assumed ±30% uncertainty in the estimates of radiative forcing of O_3_ to reflect the uncertainties in the non-linear atmospheric chemical reactions.

Based on these uncertainty ranges, we performed eight sensitivity experiments with the atmospheric chemistry model GEOS-Chem to assess how the flux uncertainties will propagate into the estimates of the Nr based radiative forcing in 2019. Specifically, we performed separate simulations for each component (NBP, N_2_O, NO_x_, NH_3_) assuming emission trajectories between 1850 and 2019 ± one standard deviation of the ensemble mean. As described in the Methods section of the main text, these emission changes were fed into GEOS-Chem either directly (NH_3_ and NO_x_) or indirectly using the atmospheric concentrations derived from the box models (CO_2_, N_2_O and CH_4_). The results from these eight GEOS-Chem sensitivity experiments, summarized in Table S1, were directly used to get the uncertainty ranges in Figs. 1 and 3.

S1.3 Present-day sensitivity of radiative forcing to Nr forcing changes

We diagnosed the sensitivity (*S_i,_* see Table S2) of radiative forcings to changes in concentrations of CO_2_, N_2_O and CH_4_ or emissions of NH_3_ and NO_x_ for present-day perturbations from the GEOS-Chem sensitivity simulations described in S 1.2 as:

$S_{i}=\frac{{RF}_{i, max}-{RF}_{i,min}}{X_{i, max}-X_{i,min}}$ (S1)

where *RF_i,max_* and *RF_i,min_* are the direct radiative forcings of the component *i* at the upper (*X_i,max_*) and lower (*X_i,min_*) atmospheric concentrations (emissions) of CO_2_, N_2_O and CH_4_ (NH_3_ and NO_x_), which were taken as inputs to the eight GEOS-Chem sensitivity experiments (Table S1). We use the *S_i_* for the analysis reported in Fig. 4, which quantifies the uncertainties in the attribution of the Nr climate effect to both agricultural or non-agricultural sources. The *S_i_* are further utilized as input for in Eqs. 6-10 (Methods) to estimate the effects of future Nr changes on the climate system, as represented in Fig. 5, where the potential uncertainties for the future extrapolation are given by utilizing the percentage ratio of RF in Fig. 3

**S2. Discussion of uncertainties and caveats**

**S2.1 Nr effects on terrestrial carbon balance**

Our estimates of anthropogenic Nr stimulation on terrestrial carbon sinks range about 0.55±0.38 Pg C yr^-1^ over 2016-2020 (Fig. 2a), which are close to previous estimates^7^ considering the continued N addition between the 2000s and the period of 2016-2020. However, discrepancies between estimates from meta-analyses and terrestrial biosphere models remain, especially when partitioning the effects into agricultural and non-agricultural contributions. Here, the NMIP2 ensemble results showed a contribution of about 0.29 Pg C yr^-1^ by fertilizer and manure applications (dominated by agricultural croplands and grassland) and of about 0.26 Pg C yr^-1^ by N deposition (dominated by forest) averaged over 2016-2020 (Fig. 2a). However, a meta-analysis study by Liu and Greaver ^8^ estimated N addition led to 1.31 ± 0.46 Pg C yr^-1^ and 0.31 ± 0.0646 Pg C yr^-1^ increases on global forest and crop carbon sequestration, respectively. Recently, with more available data, the latest meta-analyses showed much weaker effects on forest (41 (−53 to 159) Tg C yr^-1^ on global forest biomass)^9^ but stronger effects on agriculture crops^10^. It is still challenging to reconcile these estimates with current understanding of the global carbon budget^11^. More wide-spread and long-lasting ecosystem manipulation experiments as well as the further development of C-N coupling terrestrial biosphere models^12^ are both essential to fill this gap.

The estimates presented in this study do not account for the CO_2_ emissions due to artificial N fixation as part of the Haber Bosch process, because they are not directly related to the anthropogenic Nr effects, and are implicitly accounted as part of fossil-fuel based carbon emissions. Nevertheless, as a first estimate with an emission factor of 1.87 ton CO_2_ per 1 ton NH_3_ produced^13^, the cumulated fossil fuel CO_2_ emissions (C_emiss,HB_) from Haber Bosch are about 2.6 Pg C with ca. 4.021 Pg N cumulative fertilizer production over 1850-2019 (C_emiss,HB_ = 4.021 * (17.0 /14.0) * 1.87 * (12.0 / 44.0)). In comparison, this is 6.6% of the NMIP2 ensemble estimate of 36.4 Pg C cumulated NBP due to anthropogenic Nr. However, the constant or even slightly increasing fertilizer production embedded in the future SSP scenarios will result in constant or higher CO_2_ emissions from Nr generation, and thus slightly weaken the cooling effects led by N fertilization effects on terrestrial carbon sinks.

The extrapolation of the anthropogenic Nr effect into the next three decades relies on the cumulative response of the C cycle to Nr inputs over 1850-2020. This approach does not consider the potential for future N saturation in terrestrial ecosystems, and therefore potentially leads to an overestimate of the future terrestrial carbon uptake and subsequent cooling effects. Ecosystems with high anthropogenic Nr inputs (e.g. croplands; forest and grassland in dense-population regions) may already be saturated with Nr at present, as shown by previous data-based studies^14-16^ and the results of the NMIP2 ensemble (Figs. S3 and S4). To estimate the magnitude of the potential N saturation on the future climate forcing from anthropogenic Nr, we repeated the SSP-scenario experiments, but assumed that future fertilizer and manure application would not further enhance NBP. This modification, shown in Figure S5, does not change the patterns reported in Fig. 5, but exhibits a slight tendency towards stronger warming effects (0.02-0.03 W m^-2^ increases by 2050s, Table S4) when considering the N saturation effect. This demonstrates that despite uncertainties in the extend of N saturation, the key findings of our studies remain robust.

**S2.2 Global N_2_O budgets**

Our estimates based on CEDS inventory and NMIP2 ensembles were generally within the uncertainty ranges of IPCC AR6 ^11^. The CEDS inventory estimated 1.9 Tg N yr^-1^ N_2_O emissions averaged over 2016-2020 from fossil fuel combustion (Fig. 2b), which was higher than IPCC AR6 estimate of 1.0 Tg N yr^-1^ over 2007-2016 based on the EDGAR inventory^11^. Furthermore, the N_2_O emissions from open ocean were estimated by the pre-industrial equilibrium of box model and thus contained all uncertainties from other sources. Despite such simplification, our estimates of 3.3 Tg N yr^-1^ (Extended Data Table 4) were still close to the IPCC central estimate over 2007-2016^11^ (3.4 Tg N yr^-1^).

**S2.3 Uncertainties in global NH_3_ emissions**

There are significant uncertainties in the global NH_3_ budget. Bottom-up inventories have been widely reported to underestimate agricultural NH_3_ emissions especially in hotspot regions^17,18^. A recent bottom-up global assessment estimated agricultural NH_3_ emissions of 58 Tg N yr^-1^ in 2010^19^, which is substantially higher than the corresponding CEDS estimate of 35.3 Tg N yr^-1^ for the same year. Top-down methods combining satellite data and model inversions also provide a large range of estimates for the total global NH_3_ source of 64 Tg N yr^-1^ to 148 Tg N yr^-1 18,20^. The global anthropogenic NH_3_ emissions of 50.5 Tg N yr^‑1^ in 2019 used in this study are based on the original CEDS inventory (Extended Data Fig. 3), and therefore might be a somewhat low biased estimate. However, resolving these differences in NH_3_ emissions is beyond the scope of this study.

Another uncertainty of NH_3_ in this study stems from challenges in separating anthropogenic sources from the agricultural NH_3_ emissions. The fixed present-day partitioning of soil and livestock sources as 48% and 52%, respectively, ignores spatial discrepancies, which could affect the regional estimates of atmospheric NH_3_ burden. We also note the large inter-model variability within the NMIP2 ensemble could influence not only magnitude but also attribution of anthropogenic contributions of soil NH_3_ volatilization (Fig. 2d). As a result, our assessment of NH_3_ is likely the most uncertain components in our climate effect attributions.

**S2.4 Non-linear effects of NO_x_ on atmospheric chemistry**

The atmospheric oxidation capacity is very sensitive to changes in NO_x_ emissions but in a non-linear fashion. In this study, we assessed the NO_x_ effects on CH_4_ lifetime in the CH_4_ box model by one model parameter, which is insufficient to precisely capture the known non-linearity, but using the uncertainty range of ±30% covers the range of the expected non-linear effects. Besides, ignoring the spatial distributions of NO_x_ in the CH_4_ box model may lead to uncertainties in the regional quantification considering the short lifetime of NO_x_ and OH. However, this effect is likely mitigated globally by the longer lifetime of CH_4_. Furthermore, the ozone chemical mechanism between precursors of NO_x_ and volatile organic compounds (VOCs) ^21^ could make the linear extrapolation uncertain even though ozone is not the leading climate effects. Many recent studies showed that surface ozone concentrations are likely to increase in response to NO_x_ reduction especially in heavily-polluted VOCs-limited regions ^22^, for example in metropolitan area during the COVID-19 lockdown period ^23,24^, but the climate effect of such surface changes will be attenuated by the column-integrated radiative effect of tropospheric ozone.

**S2.5 Missing processes in this study**

Given the limitations of the biosphere models used in this study, the influence of aerosols or surface O_3_ on terrestrial carbon fluxes were ignored due to the large uncertainties associated with their quantification. Previous studies showed that the increased diffuse radiation due to increased aerosol loadings could facilitate plant carbon assimilation^25^; while O_3_ exposure commonly leads to plant foliage damage and reduced plant productivity^26^. However, the magnitudes of these effects were widely varying across different studies^26-28^. We also note that most of the aforementioned studies only focused on the effects on plant productivity, but rarely on the net ecosystem carbon fluxes, which are relevant for our study. IPCC AR6 suggested that there was already ‘robust evidences’ to represent these effects^2^ but quantifying the aerosol and O_3_ effects on terrestrial carbon cycles remained ‘uncertain’ with ‘low confidence’. The omission of these effects implys that the cooling effects by aerosols as well as the warming effects due to O_3_ as represented in this study may be both underestimated.

One important limitation of our analysis with GEOS-Chem is that it does not include the aerosol indirect effects (aerosol-cloud interactions). The set-up of GEOS-Chem, driven by reanalyzed meteorology with prescribed cloudiness, makes the assessment of aerosol-cloud interactions impossible. Nevertheless, the indirect climate effects of aerosols remain the largest uncertainties among all of the short-lived climate forcing^2^. Some studies used to estimate much stronger cooling effects of nitrate-cloud interactions relative to the nitrate direct diffusing effects^29^. Further studies including an interactive cloud scheme would be required to quantify the magnitude of the Nr aerosol effects on cloud at a global scale, but this is beyond the scope of this study.

Some studies have reported that Nr addition could reduce soil CH_4_ uptake^8,30,31^. One study estimated that the global Nr-induced reduction in soil CH_4_ uptake was estimated as 2.9–6.9 Tg CH_4_ yr^-1 8^, which is a minor fraction of terrestrial emissions in the global CH_4_ budget^32^. However, there is significant ambiguity in the field-based evidence for such an effect. For example, Xia, et al. ^33^ found a shift from a positive to a negative effect on soil CH_4_ uptake with increasing N additions in boreal and temperate forest. Furthermore, few terrestrial biosphere models with C-N dynamics in NMIP2 have the capability to simulate CH_4_ and the Nr effects on soil CH_4_ emissions. As a result, this effect was not included in our study.

In this study, the marine Nr emissions were all assumed to be from natural sources and not significantly affected by anthropogenic Nr. IPCC AR6^11^ reported that oceanic N deposition could increase N_2_O emissions by 0.01–0.32 Tg N yr^–1^ , which is a minor effect compared to the uncertainties in marine N_2_O emissions (2.5-4.3 Tg N yr^-1^ in IPCC AR6). The marine biogeochemical NO_x_ and NH_3_ emissions were estimated by 7.3-10.3 Tg N yr^-1^ ^34^and 2.5-23 Tg N yr^-1 35^, respectively, but both are still poorly understood and usually accounted as natural sources. Although some studies reported anthropogenic Nr deposition could enhance global ocean primary productivity by about 0.3 Pg C yr^-1 36^, the net effect on marine net carbon flux is still unclear. Nutrient effects on the ocean carbon cycles are generally attributed to the upwelling of subsurface water rather than the anthropogenic Nr deposition although riverine transport of anthropogenic Nr may play a role regionally. In summary, Nr-related marine emissions are considered to be of low magnitude relative to the sources of fossil fuel combustion or land-based fluxes, but more observations are needed to better characterized these sources to fill the knowledge gap.

**S3. Comparison to previous studies**

To our knowledge, there was only one review study by Erisman, et al. ^37^ which estimated the net radiative forcing from anthropogenic Nr. However, this study was based on a literature review and did not assess the combined and partially interactive effect of Nr in the atmosphere as we have done in our study. More importantly, the uncertainty ranges in Erisman, et al. ^37^ were just derived based on estimates from Europe. With these limitations, they estimated a net cooling effects of -0.24 W m^-2^ with ranges from +0.2 to -0.5 W m^-2^, which is of comparable magnitude but with a much larger uncertainty range and subject to more implicit uncertainty relative to our estimate (-0.34 [-0.20 to -0.50] W m^-2^).

Our estimates of radiative forcing compare favorably with previous studies regarding each separate component (Table S3). In general, our estimates fit well in the uncertainty ranges from previous studies. In such comparisons, it should also be noted that different studies may use different data, different reference year, or different definition of anthropogenic Nr, and thus resulting in difficulties in synthesizing the net climate effects of anthropogenic Nr. The large discrepancies among different studies on different components were exactly one of the important reasons that motivated us to design such a uniformed framework in this study.

**References**

1 Tian, H. *et al.* Global Nitrous Oxide Budget 1980-2020. *Earth Syst. Sci. Data Discuss.* **2023**, 1-98, doi:10.5194/essd-2023-401 (2023).

2 Szopa, S., V. *et al.* Short-Lived Climate Forcers. In Climate Change 2021: The Physical Science Basis. Contribution of Working Group I to the Sixth Assessment Report of the Intergovernmental Panel on Climate Change. 817–922 (2021).

3 Jena, C. *et al.* Inter-comparison of different NOX emission inventories and associated variation in simulated surface ozone in Indian region. *Atmospheric Environment* **117**, 61-73, doi:<https://doi.org/10.1016/j.atmosenv.2015.06.057> (2015).

4 Ding, J. *et al.* Intercomparison of NOx emission inventories over East Asia. *Atmos. Chem. Phys.* **17**, 10125-10141, doi:10.5194/acp-17-10125-2017 (2017).

5 McDuffie, E. E. *et al.* A global anthropogenic emission inventory of atmospheric pollutants from sector- and fuel-specific sources (1970-2017): an application of the Community Emissions Data System (CEDS). *Earth System Science Data* **12**, 3413-3442, doi:10.5194/essd-12-3413-2020 (2020).

6 Murray, L. T., Fiore, A. M., Shindell, D. T., Naik, V. & Horowitz, L. W. Large uncertainties in global hydroxyl projections tied to fate of reactive nitrogen and carbon. *Proceedings of the National Academy of Sciences of the United States of America* **118**, doi:10.1073/pnas.2115204118 (2021).

7 Zaehle, S., Ciais, P., Friend, A. D. & Prieur, V. Carbon benefits of anthropogenic reactive nitrogen offset by nitrous oxide emissions. *Nature Geoscience* **4**, 601-605, doi:10.1038/ngeo1207 (2011).

8 Liu, L. L. & Greaver, T. L. A review of nitrogen enrichment effects on three biogenic GHGs: the CO2 sink may be largely offset by stimulated N2O and CH4 emission. *Ecology Letters* **12**, 1103-1117, doi:10.1111/j.1461-0248.2009.01351.x (2009).

9 Schulte-Uebbing, L. F., Ros, G. H. & de Vries, W. Experimental evidence shows minor contribution of nitrogen deposition to global forest carbon sequestration. *Global Change Biology* **28**, 899-917, doi:10.1111/gcb.15960 (2022).

10 Lessmann, M., Ros, G. H., Young, M. D. & de Vries, W. Global variation in soil carbon sequestration potential through improved cropland management. *Global Change Biology* **28**, 1162-1177, doi:10.1111/gcb.15954 (2022).

11 Canadell.J.G *et al.* Global Carbon and other Biogeochemical Cycles and Feedbacks. In Climate Change 2021: The Physical Science Basis. Contribution of Working Group I to the Sixth Assessment Report of the Intergovernmental Panel on Climate Change. 673–816 (2021).

12 Kou-Giesbrecht, S. *et al.* Evaluating nitrogen cycling in terrestrial biosphere models: a disconnect between the carbon and nitrogen cycles. *Earth Syst. Dynam.* **14**, 767-795, doi:10.5194/esd-14-767-2023 (2023).

13 Wang, Y. & Meyer, T. J. A Route to Renewable Energy Triggered by the Haber-Bosch Process. *Chem* **5**, 496-497, doi:10.1016/j.chempr.2019.02.021 (2019).

14 Peng, Y. F., Chen, H. Y. H. & Yang, Y. H. Global pattern and drivers of nitrogen saturation threshold of grassland productivity. *Functional Ecology* **34**, 1979-1990, doi:10.1111/1365-2435.13622 (2020).

15 He, N. P. *et al.* Global patterns of nitrogen saturation in forests. *Pre-print*, doi:<https://doi.org/10.21203/rs.3.rs-3559857/v1> (2023).

16 Schulte-Uebbing, L. F., Beusen, A. H. W., Bouwman, A. F. & de Vries, W. From planetary to regional boundaries for agricultural nitrogen pollution. *Nature* **610**, 507-+, doi:10.1038/s41586-022-05158-2 (2022).

17 Zhang, X. M. *et al.* Ammonia Emissions May Be Substantially Underestimated in China. *Environmental Science & Technology* **51**, 12089-12096, doi:10.1021/acs.est.7b02171 (2017).

18 Luo, Z. *et al.* Estimating global ammonia (NH3) emissions based on IASI observations from 2008 to 2018. *Atmos. Chem. Phys.* **22**, 10375-10388, doi:10.5194/acp-22-10375-2022 (2022).

19 Liu, L. *et al.* Exploring global changes in agricultural ammonia emissions and their contribution to nitrogen deposition since 1980. *Proceedings of the National Academy of Sciences of the United States of America* **119**, doi:10.1073/pnas.2121998119 (2022).

20 Evangeliou, N. *et al.* 10-year satellite-constrained fluxes of ammonia improve performance of chemistry transport models. *Atmos. Chem. Phys.* **21**, 4431-4451, doi:10.5194/acp-21-4431-2021 (2021).

21 Ren, J., Guo, F. F. & Xie, S. D. Diagnosing ozone-NOx-VOC sensitivity and revealing causes of ozoneincreases in China based on 2013-2021 satellite retrievals. *Atmospheric Chemistry and Physics* **22**, 15035-15047, doi:10.5194/acp-22-15035-2022 (2022).

22 Simon, H., Reff, A., Wells, B., Xing, J. & Frank, N. Ozone Trends Across the United States over a Period of Decreasing NOx and VOC Emissions. *Environmental Science & Technology* **49**, 186-195, doi:10.1021/es504514z (2015).

23 Huang, X. *et al.* Enhanced secondary pollution offset reduction of primary emissions during COVID-19 lockdown in China. *National Science Review* **8**, doi:10.1093/nsr/nwaa137 (2021).

24 Zhang, K. *et al.* Insights into the significant increase in ozone during COVID-19 in a typical urban city of China. *Atmospheric Chemistry and Physics* **22**, 4853-4866, doi:10.5194/acp-22-4853-2022 (2022).

25 Mercado, L. M. *et al.* Impact of changes in diffuse radiation on the global land carbon sink. *Nature* **458**, 1014-U1087, doi:10.1038/nature07949 (2009).

26 Sitch, S., Cox, P. M., Collins, W. J. & Huntingford, C. Indirect radiative forcing of climate change through ozone effects on the land-carbon sink. *Nature* **448**, 791-U794, doi:10.1038/nature06059 (2007).

27 Lombardozzi, D., Levis, S., Bonan, G. & Sparks, J. P. Predicting photosynthesis and transpiration responses to ozone: decoupling modeled photosynthesis and stomatal conductance. *Biogeosciences* **9**, 3113-3130, doi:10.5194/bg-9-3113-2012 (2012).

28 Yue, X. & Unger, N. The Yale Interactive terrestrial Biosphere model version 1.0: description, evaluation and implementation into NASA GISS ModelE2. *Geoscientific Model Development* **8**, 2399-2417, doi:10.5194/gmd-8-2399-2015 (2015).

29 Wu, M. *et al.* Development and Evaluation of E3SM-MOSAIC: Spatial Distributions and Radiative Effects of Nitrate Aerosol. *Journal of Advances in Modeling Earth Systems* **14**, e2022MS003157, doi:<https://doi.org/10.1029/2022MS003157> (2022).

30 Steudler, P. A., Bowden, R. D., Melillo, J. M. & Aber, J. D. INFLUENCE OF NITROGEN-FERTILIZATION ON METHANE UPTAKE IN TEMPERATE FOREST SOILS. *Nature* **341**, 314-316, doi:10.1038/341314a0 (1989).

31 Li, Q., Peng, C. H., Zhang, J. B., Li, Y. F. & Song, X. Z. Nitrogen addition decreases methane uptake caused by methanotroph and methanogen imbalances in a Moso bamboo forest. *Scientific Reports* **11**, doi:10.1038/s41598-021-84422-3 (2021).

32 Saunois, M. *et al.* The Global Methane Budget 2000-2017. *Earth System Science Data* **12**, 1561-1623, doi:10.5194/essd-12-1561-2020 (2020).

33 Xia, N. *et al.* Effects of nitrogen addition on soil methane uptake in global forest biomes. *Environmental Pollution* **264**, doi:10.1016/j.envpol.2020.114751 (2020).

34 Song, W., Liu, X. Y., Houlton, B. Z. & Liu, C. Q. Isotopic constraints confirm the significant role of microbial nitrogen oxides emissions from the land and ocean environment. *National Science Review* **9**, doi:10.1093/nsr/nwac106 (2022).

35 Paulot, F. *et al.* Global oceanic emission of ammonia: Constraints from seawater and atmospheric observations. *Global Biogeochemical Cycles* **29**, 1165-1178, doi:10.1002/2015gb005106 (2015).

36 Duce, R. A. *et al.* Impacts of atmospheric anthropogenic nitrogen on the open ocean. *Science* **320**, 893-897, doi:10.1126/science.1150369 (2008).

37 Erisman, J. W., Galloway, J., Seitzinger, S., Bleeker, A. & Butterbach-Bahl, K. Reactive nitrogen in the environment and its effect on climate change. *Current Opinion in Environmental Sustainability* **3**, 281-290, doi:10.1016/j.cosust.2011.08.012 (2011).

**Table S1. Inputs for the eight GEOS-Chem sensitivity experiments.** All of the eight experiments follow the No_allNr experiment but just change one single compound based on the standard deviation of NMIP2 ensembles. The NO_x_ and NH_3_ emissions are directly from the upper and lower boundaries in Fig. S2, while the greenhouse (CO_2_, N_2_O and CH_4_) concentrations are derived by box models for each species (See Methods). Values in brackets denote the reference value from the 2019_CTRL experiment ± the change in the sensitivity experiment.

| Exp_name | CO_2_ Conc. (ppmv) | N_2_O Conc. (ppbv) | CH_4_ Conc.^*^ (ppbv) | NO_x_ emissions (Tg N yr^-1^) | NH_3_ emissions^**^ (Tg N yr^-1^) |
| --- | --- | --- | --- | --- | --- |
| CO_2__min | 414.27 ppmv  (409.9+4.37) | 286.33 ppbv  (326.53-40.20) | 2354.16 ppbv  (2015.02+339.14) | 9.14 | 9.16 |
| CO_2__max | 426.48 ppmv  (409.9+16.58) | 286.33 ppbv  (326.53-40.20) | 2354.16 ppbv  (2015.02+339.14) | 9.14 | 9.16 |
| N_2_O_min | 420.38 ppmv  (409.9+10.48) | 292.49 ppbv  (326.53-34.04) | 2354.16 ppbv  (2015.02+339.14) | 9.14 | 9.16 |
| N_2_O_max | 420.38 ppmv  (409.9+10.48) | 280.17 ppbv  (326.53-46.36) | 2354.16 ppbv  (2015.02+339.14) | 9.14 | 9.16 |
| CH_4__min | 420.38 ppmv  (409.9+10.48) | 286.33 ppbv  (326.53-40.20) | 2093.24 ppbv  (2015.02+78.22) | 9.14 | 9.16 |
| CH_4__max | 420.38 ppmv  (409.9+10.48) | 286.33 ppbv  (326.53-40.20) | 2737.18 ppbv  (2015.02+722.16) | 9.14 | 9.16 |
| Aerosol_min | 420.38 ppmv  (409.9+10.48) | 286.33 ppbv  (326.53-40.20) | 2354.16 ppbv  (2015.02+339.14) | 9.82 | 27.14 |
| Aerosol_max | 420.38 ppmv  (409.9+10.48) | 286.33 ppbv  (326.53-40.20) | 2354.16 ppbv  (2015.02+339.14) | 8.45 | 0.0 |

^*^Uncertainty in CH_4_ concentrations were dominant by the estimated 30% uncertainties in global OH concentrations rather than the relatively small uncertainties in NOx emissions.

^**^Due to the large uncertainty in estimating global NH_3_ emissions, as we also demonstrated in the main text, the upper boundary of anthropogenic contributions on NH_3_ (62.2 Tg N yr^-1^ in 2019) have already exceed our current global total estimates (50.5 Tg N yr^-1^ in 2019). As a result, we assumed the global NH_3_ anthropogenic emissions to be zero in the sensitive experiments as the guess of maximum contribution of anthropogenic Nr on NH_3_.

**Table S2. Sensitivity of RF to changes in greenhouse gas concentrations or Nr precursors’ emissions**. The values are calculated from the simulations described in S1.3. *S_i_* is defined in equation S1.

|  | CO_2_  (W m^-2^ ppmv^-1^) | N_2_O  (W m^-2^ ppbv^-1^) | CH_4_  (W m^-2^ ppbv^-1^) | Aerosol  (W m^-2^ (Tg N)^-1^) |
| --- | --- | --- | --- | --- |
| *S_i_* | -0.00819 | 0.00244 | 0.00026 | -0.0035 |

**Table S3. Comparison of the radiative forcing induced by individual Nr component or process between previous independent studies and the estimates in this study.** The uncertainty ranges in previous studies were either based on one standard deviation of forcing data (terrestrial carbon sinks) and model ensembles (N_2_O and O_3_), or indicate the ranges among different models or studies (NO_x_ effects on CH_4_ and aerosol effects)

|  | Previous study | This study |
| --- | --- | --- |
| Terrestrial carbon sinks | -0.096±0.014 W m^-2^  (until 2005) ^7^ | -0.12 [-0.17 -0.07] W m^-2^  (until 2019) |
| Atmospheric N_2_O | +0.21±0.03 W m^-2 38^  (all N_2_O emission factors) | +0.16 [+0.14, + 0.17] W m^-2^  (only fertilizer, manure, N deposition and fossil fuel) |
| Atmospheric CH_4_ through NO_x_ perturbating OH | [-0.38, -0.2] W m^-2 39^ | -0.19 [-0.28, -0.11] W m^-2^ |
| Ammonium aerosols (direct effects) | [-0.12, -0.07] W m^-2 39,40^ | -0.24 [-0.28, -0.18] W m^-2^ |
| Nitrate aerosols (direct effects) | [-0.14, -0.025] W m^-2 39,41-44^ |  |
| O_3_ due to anthropogenic NO_x_ | +0.2±0.07 W m^-2 39^  (in 2014; under pre-industrial VOC levels) | +0.05 [+0.03, +0.07] W m^-2^  (in 2019; under present-day VOC levels) |

References in Table S3

7 Zaehle, S., Ciais, P., Friend, A. D. & Prieur, V. Carbon benefits of anthropogenic reactive nitrogen offset by nitrous oxide emissions. *Nature Geoscience* **4**, 601-605, doi:10.1038/ngeo1207 (2011).

38 Forster, P. *et al.* The Earth’s Energy Budget, Climate Feedbacks, and Climate Sensitivity. In Climate Change 2021: The Physical Science Basis. Contribution of Working Group I to the Sixth Assessment Report of the Intergovernmental Panel on Climate Change. 923–1054 (2021).

39 Thornhill, G. D. *et al.* Effective radiative forcing from emissions of reactive gases and aerosols - a multi-model comparison. *Atmospheric Chemistry and Physics* **21**, 853-874, doi:10.5194/acp-21-853-2021 (2021).

40 Heald, C. L. *et al.* Contrasting the direct radiative effect and direct radiative forcing of aerosols. *Atmospheric Chemistry and Physics* **14**, 5513-5527, doi:10.5194/acp-14-5513-2014 (2014).

41 Hauglustaine, D. A., Balkanski, Y. & Schulz, M. A global model simulation of present and future nitrate aerosols and their direct radiative forcing of climate. *Atmospheric Chemistry and Physics* **14**, 11031-11063, doi:10.5194/acp-14-11031-2014 (2014).

42 Bian, H. S. *et al.* Investigation of global particulate nitrate from the AeroCom phase III experiment. *Atmospheric Chemistry and Physics* **17**, 12911-12940, doi:10.5194/acp-17-12911-2017 (2017).

43 An, Q. *et al.* The Development of an Atmospheric Aerosol/Chemistry-Climate Model, BCC_AGCM_CUACE2.0, and Simulated Effective Radiative Forcing of Nitrate Aerosols. *Journal of Advances in Modeling Earth Systems* **11**, 3816-3835, doi:10.1029/2019ms001622 (2019).

44 Zaveri, R. A. *et al.* Development and Evaluation of Chemistry-Aerosol-Climate Model CAM5-Chem-MAM7-MOSAIC: Global Atmospheric Distribution and Radiative Effects of Nitrate Aerosol. *Journal of Advances in Modeling Earth Systems* **13**, doi:10.1029/2020ms002346 (2021).

**Table S4. The accumulated NBP fluxes over 2020 to 2050 as well as the corresponding predicted radiative forcing relative to 1850 under two sensitivity future experiments under three SSP scenarios.** The present-day values are also given as a reference.

|  |  | Accumulated NBP over 2020-2050 (Pg C) | RF (CO_2_) induced by anthropogenic Nr relative to 1850 (W m^-2^) |
| --- | --- | --- | --- |
| Present day (2019) |  | 36.40  (1850-2019) | -0.121 |
| SSP 1-2.6 | With fertilizer and manure effects on NBP | 15.20 | -0.156 |
|  | Without fertilizer and manure effects on NBP | 6.90 | -0.136 |
| SSP 3-7.0 | With fertilizer and manure effects on NBP | 18.62 | -0.164 |
|  | Without fertilizer and manure effects on NBP | 8.18 | -0.139 |
| SSP 5-8.5 | With fertilizer and manure effects on NBP | 16.92 | -0.160 |
|  | Without fertilizer and manure effects on NBP | 8.17 | -0.139 |

**Table S5. The global direct radiative forcing (W m^-2^) induced by all anthrophonic Nr and the sum of agricultural and non-agricultural Nr sources.**

| Radiative forcing |  | All anthropogenic Nr (from Fig. 3) | Sum of agricultural and non-agricultural Nr | Agricultural Nr | Non-agricultural Nr |
| --- | --- | --- | --- | --- | --- |
| CO_2_ |  | -0.12 | -0.12 | -0.06 | -0.06 |
| N_2_O |  | 0.16 | 0.16 | 0.05 | 0.11 |
| CH_4_ |  | -0.19 | -0.18 | -0.04 | -0.14 |
| Aerosols | NH_4_^+^ | -0.08 | -0.08 | -0.05 | -0.03 |
|  | NO_3_^-^ | -0.13 | -0.19 | -0.09 | -0.10 |
|  | SO_4_^2-^ | -0.03 | -0.02 | 0.00 | -0.02 |
| O_3_ |  | 0.05 | 0.05 | 0.00 | 0.05 |
| Total |  | -0.34 | -0.38 | -0.19 | -0.19 |


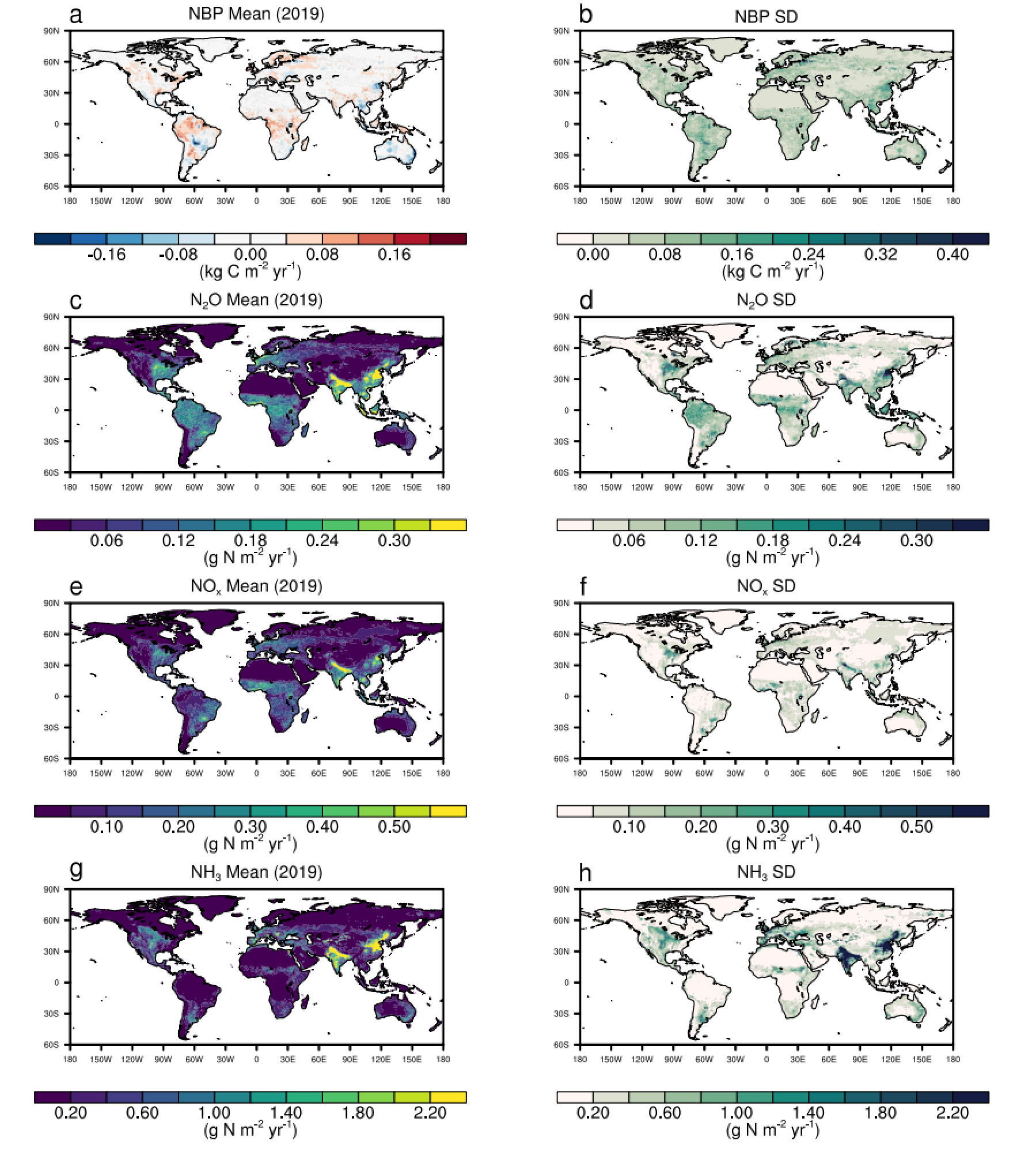


**Figure S1. Global spatial pattern of the NMIP2 the ensemble mean.** The terrestrial fluxes in 2019 as well as the standard deviation (SD) among individual models are represented for **a-b** NBP, **c-d** N_2_O, **e-f** NO_x_ and **g-h** NH_3_.


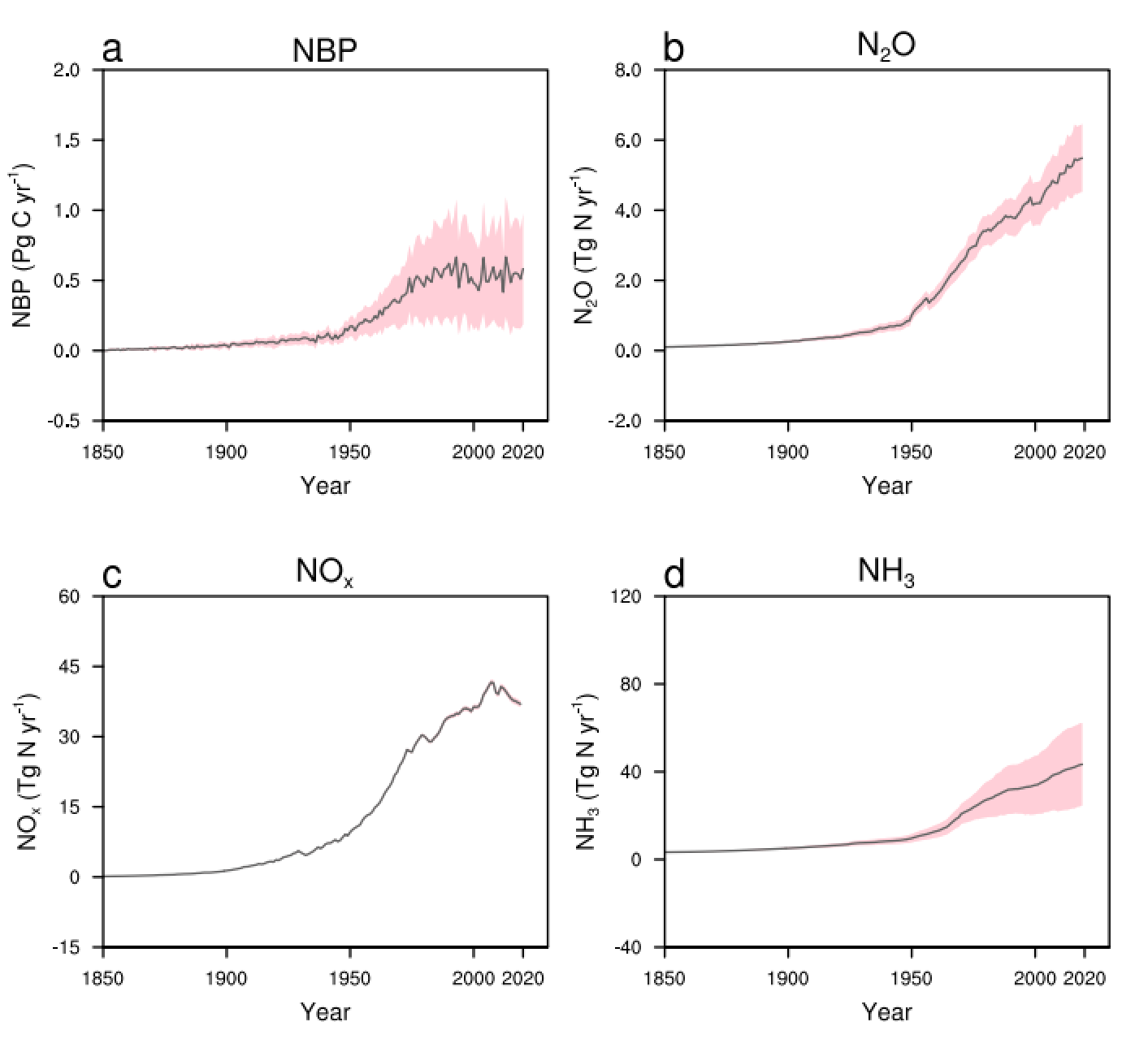


**Figure S2. Uncertainty of the anthropogenic Nr effects on historical time series.** Historical anthropogenic Nr contributions, which are defined as the sum of fertilizer and manure application, N deposition and fossil fuel combustion, on **a** NBP, **b** N_2_O emissions, **c** NO_x_ emissions and **d** NH_3_ emissions are given by black lines. The pink shades indicate the uncertainties derived by the one standard deviation among NMIP2 model ensembles.


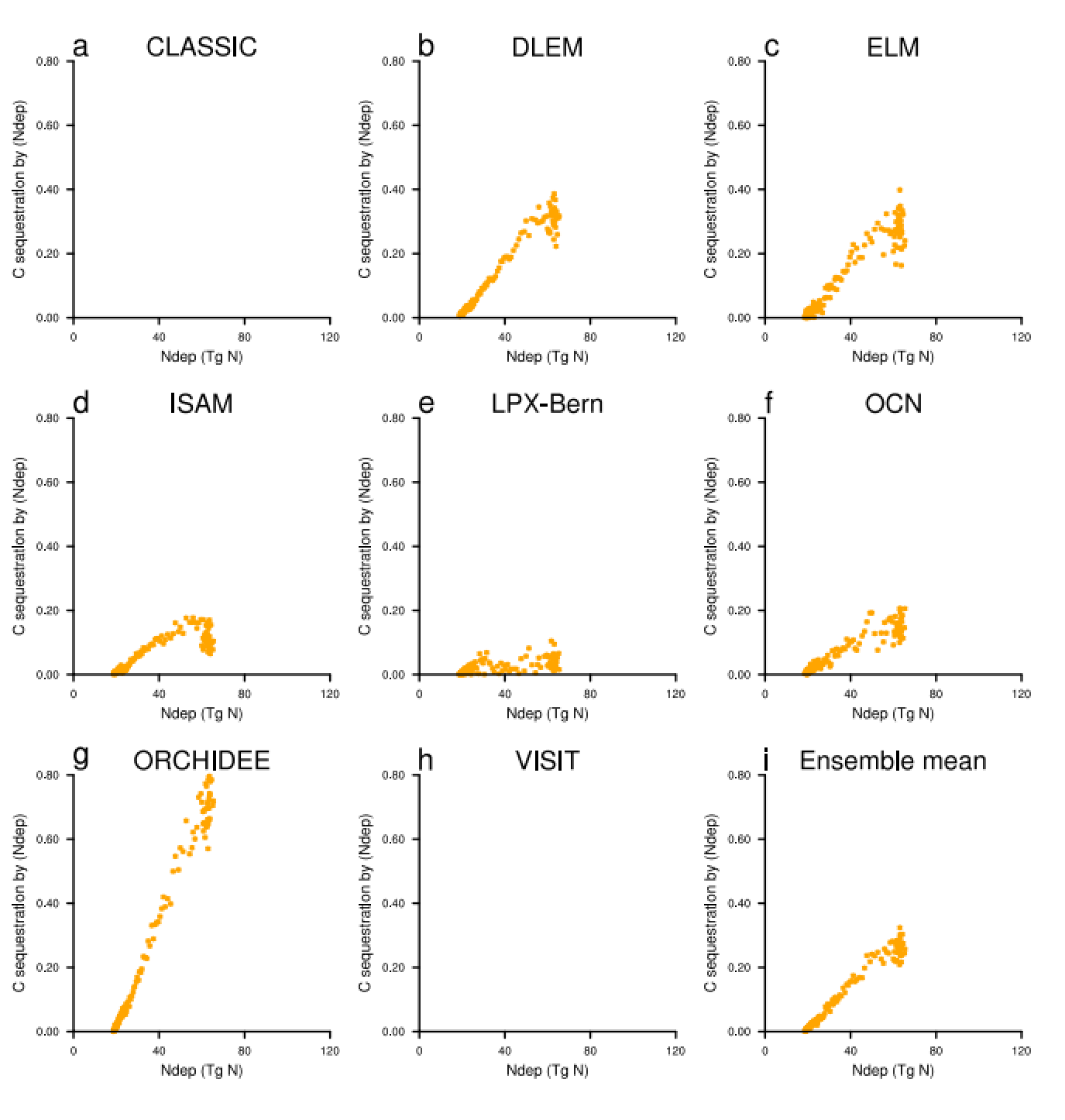


**Figure S3. Development of global net biome productivity (NBP) as a function of N deposition**. Each dot indicates the annual value of global N deposition (X axis) and global NBP enhancement (Y axis) for **a-h** each model member as well as **i** the ensemble mean from 1850 to 2019.


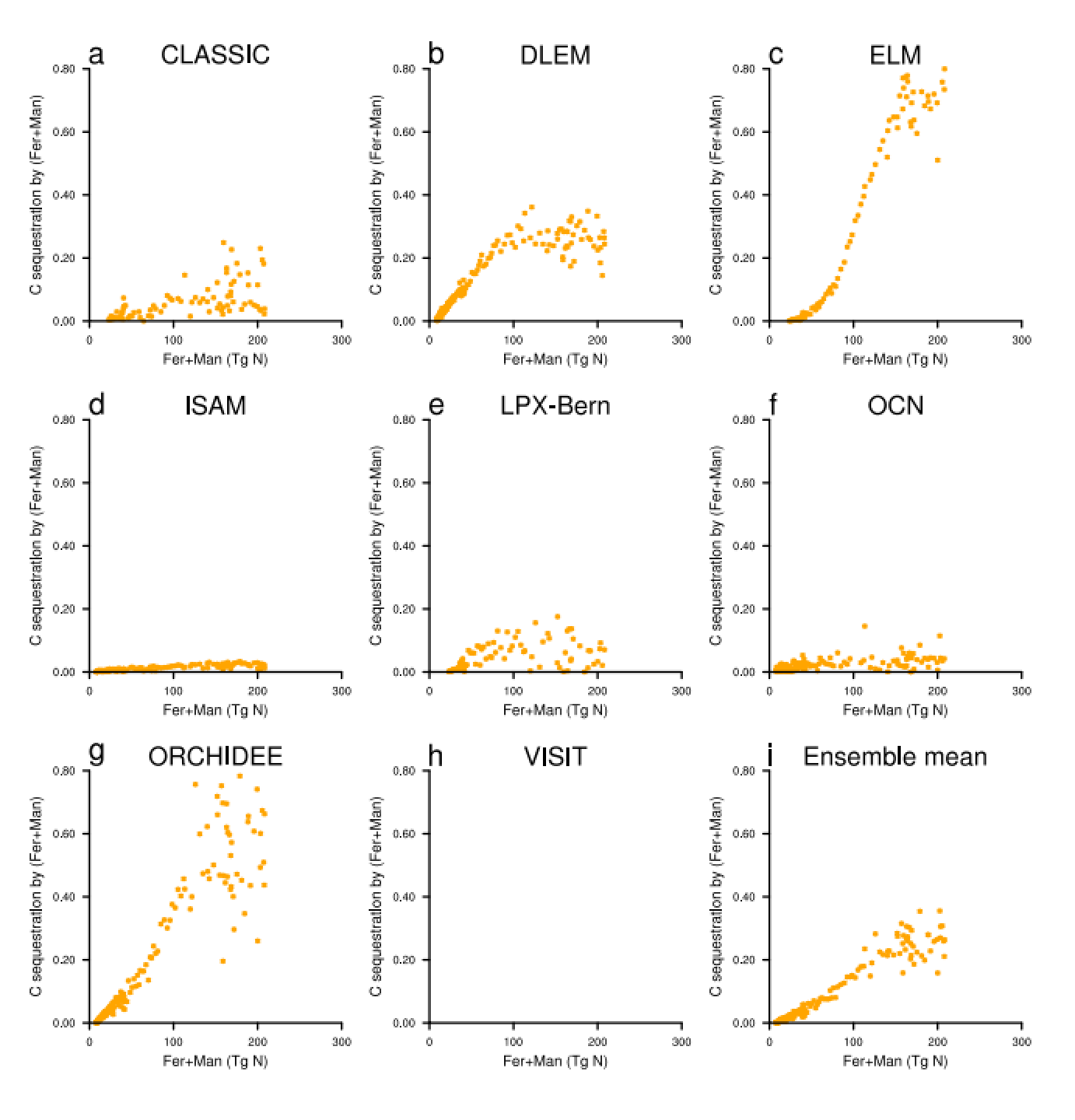


**Figure S4. Development of global net biome productivity (NBP) as a function of fertilizer and manure application**. Each dot indicates the annual value of global fertilizer and manure N application (X axis) and global NBP enhancement (Y axis) for **a-h** each model member as well as **i** the ensemble mean from 1850 to 2019.


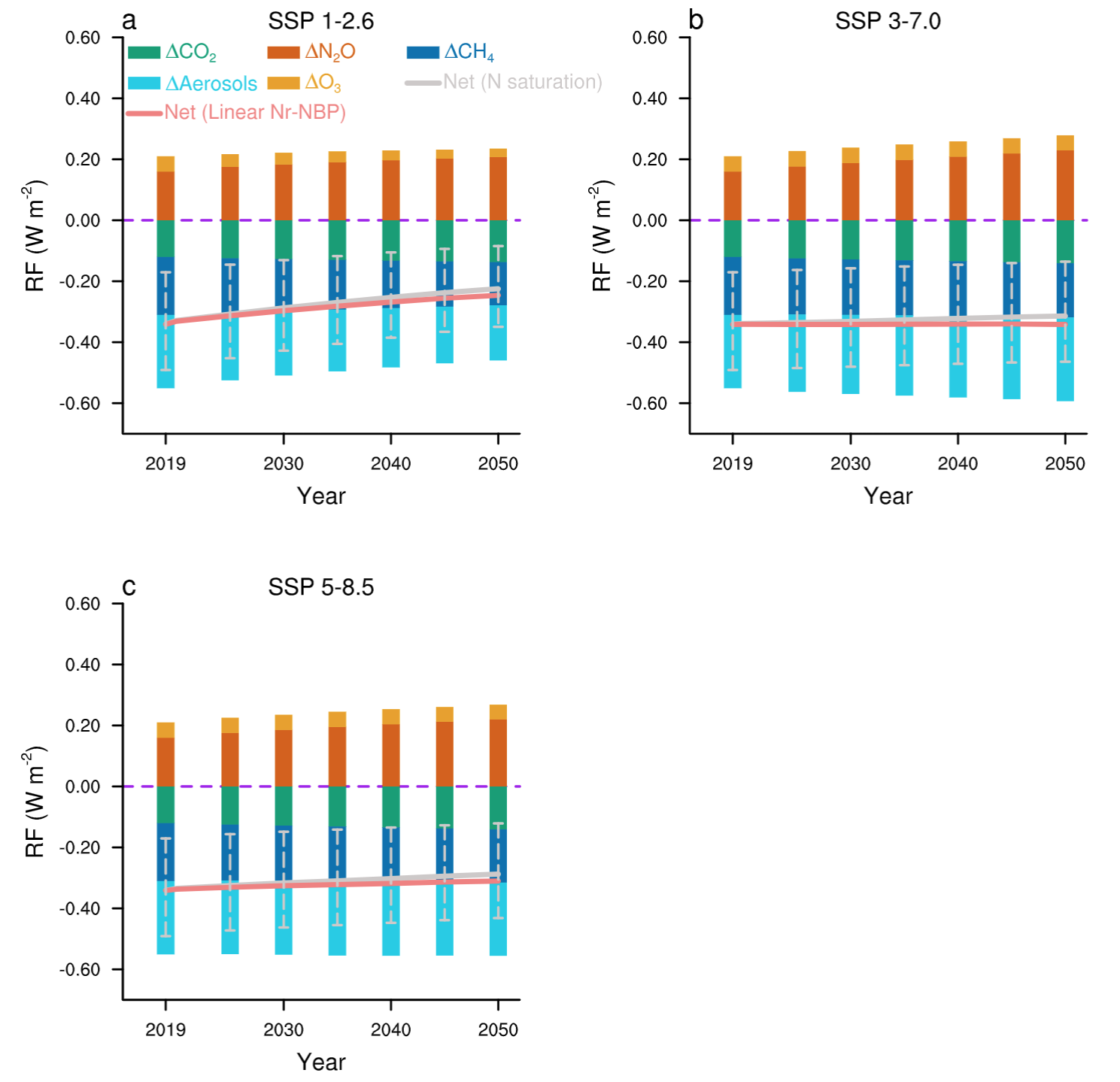


**Figure S5. Global direct radiative forcings induced by future changes in anthropogenic Nr**. Two estimates are shown with exclusion (silver lines) and inclusion (orange lines) of the effects of fertilizer and manure application on NBP, respectively. Every other forcing identically varies following the **a** SSP 1-2.6, **b** SSP 3-7.0 and **c** SSP 5-8.5 scenarios. The error bars were calculated by the percentage ranges in direct radiative forcing derived from the historical estimates (SI text S1.3)
